# A Lightweight Multi-Sensor Feature-Level Fusion Framework for Noise-Robust Fault Diagnosis of High-Precision Milling Machines

**DOI:** 10.3390/s26206449

**Published:** 2026-10-12

**Authors:** Zhi Xu, Ningyun Lu, Liguo Jin

**Affiliations:** 1 College of Automation Engineering, Nanjing University of Aeronautics and Astronautics, Nanjing 210016, China; njxuzhi@nuaa.edu.cn (Z.X.); jinliguo@nuaa.edu.cn (L.J.); 2 School of Mechanical and Electrical Engineering, Lianyungang Technical College, Lianyungang 222000, China

**Keywords:** multi-sensor fusion, feature-level fusion, milling-machine fault diagnosis, edge computing, lightweight neural network

## Abstract

The operational reliability of milling machines is a critical factor affecting manufacturing quality and productivity in modern industrial systems. Although multi-sensor fusion can overcome the limitations of single-sensor monitoring, many existing methods rely on computationally intensive deep learning models, which restrict their applicability in resource-constrained industrial environments. To address this issue, this paper presents a multi-sensor feature-level fusion framework for fault diagnosis in high-precision milling machines. The proposed framework integrates vibration signals from three sensing directions through signal preprocessing, multi-domain feature extraction, random-forest-based feature selection, feature-level fusion, and lightweight neural classification. Within this framework, a lightweight multilayer perceptron (MLP) is adopted as the default classifier to achieve an effective balance between diagnostic performance and computational efficiency for edge-oriented deployment. The experiments consider five machine states, comprising two non-fault operating states (No_Load and Normal) and three abnormal or fault-related states (Eccentricity, Bearing_Housing_Shim_15, and Bearing_Housing_Loose). Experimental results show that the proposed framework substantially outperforms single-sensor approaches, improving diagnostic accuracy from 74.00–86.00% to 96.00%. Comparative analysis across different classifier backbones further demonstrates that the lightweight MLP provides the most favorable efficiency-performance trade-off, while the multi-domain fusion (MDF) mechanism exhibits the strongest robustness under noisy conditions, maintaining 94.38 ± 1.96% accuracy at 0 dB SNR and 88.88 ± 2.53% at −10 dB SNR. These results indicate that accurate, efficient, and noise-robust milling-machine fault diagnosis can be achieved through a coordinated combination of multi-sensor feature fusion, lightweight classification, and robust fusion design, thereby providing a practical solution for intelligent industrial condition monitoring.

## 1. Introduction

The reliable operation of milling machines is essential for maintaining production efficiency, machining quality, and operational safety in modern manufacturing systems. In practical industrial environments, faults in key milling-machine components may lead to unexpected downtime, reduced product quality, and increased maintenance cost. These risks become even more significant in high-precision CNC machining, where the complexity of the machining process and the sensitivity of the equipment make early fault detection particularly important [1,2,3,4]. Therefore, intelligent and reliable fault diagnosis has become a critical requirement for advanced milling-machine monitoring and maintenance.

Traditional fault diagnosis methods for milling machines mainly rely on single-sensor monitoring, most commonly using vibration signals measured at a single location [5,6]. Although such methods can be effective for specific fault types under controlled laboratory conditions, their practical application remains limited. A single sensor provides only local dynamic information and may fail to capture fault signatures that are more pronounced at other structural locations or along different sensing directions [7,8,9]. Moreover, vibration signals acquired in real industrial environments are often contaminated by background vibration, electromagnetic interference, and measurement artifacts, resulting in low signal-to-noise ratios and reduced diagnostic reliability. In addition, different mechanical faults may produce partially similar vibration patterns when observed from only one position, increasing the risk of diagnostic ambiguity and misclassification. These limitations indicate that single-sensor monitoring is often insufficient for robust milling-machine fault diagnosis.

To overcome these limitations, fault diagnosis has gradually evolved from single-sensor monitoring to multi-sensor fusion [10,11]. Existing fusion methods can generally be categorized into data-level, feature-level, and decision-level fusion, each with different advantages and limitations [12,13,14,15]. Among these strategies, feature-level fusion provides an effective balance between information richness and computational efficiency, making it especially suitable for industrial diagnosis tasks. At the same time, deep learning has shown strong capability in automatic representation learning and fault classification [16,17,18,19,20,21,22]. However, many high-performing deep architectures rely on large parameter counts and intensive computations, which restrict their deployment on resource-constrained industrial platforms and edge devices [23,24]. As a result, although multi-sensor deep diagnosis has achieved promising performance, the combined challenge of robust information integration and efficient deployment remains insufficiently addressed.

### 1.1. Fault Characteristics and Diagnostic Relevance

Fault diagnosis in milling machines is fundamentally linked to dynamic response variations induced by different abnormal operating conditions. In practical machining systems, faults related to the spindle, cutting tool, bearings, and transmission components may lead to distinct changes in vibration amplitude, spectral distribution, impulsive behavior, and time-frequency energy concentration [25,26]. These fault-induced responses are often nonlinear, spatially heterogeneous, and component-dependent, making accurate identification difficult when relying on a single signal representation. Therefore, an effective diagnosis framework should be capable of capturing both global statistical characteristics and localized transient patterns [27].

Different machine states in the milling-machine system may exhibit markedly different vibration signatures. Relatively stable operating conditions, such as normal or no-load states, usually present smoother signal evolution and weaker impulsive components, whereas structural or rotating faults may introduce amplitude modulation, spectral redistribution, local resonance variation, and transient burst-like responses [28]. In particular, eccentricity-related faults are more likely to produce periodic fluctuation and frequency-domain anomalies, while bearing-housing-related faults tend to generate stronger impact behavior and more complex time-frequency irregularity. These differences indicate that the diagnostic information associated with different machine states and abnormal conditions is distributed across multiple signal perspectives rather than concentrated in a single feature space.

From the perspective of signal representation, different feature domains provide complementary diagnostic information. Time-domain features are suitable for characterizing overall amplitude fluctuation, dispersion, and impulsiveness; frequency-domain features are effective for identifying periodic fault-induced spectral components and energy redistribution; and time-frequency features can reveal transient or non-stationary behavior that is difficult to capture in purely stationary analysis. Therefore, the physical diversity of milling-machine fault responses directly motivates the extraction of vibration features in the time, frequency, and wavelet domains. This multi-domain feature strategy enables the diagnostic framework to preserve complementary fault evidence from different signal perspectives and establishes the physical basis for the feature selection and fusion procedures introduced in the following sections.

### 1.2. Multi-Sensor Diagnosis and Feature-Level Fusion

Compared with single-sensor monitoring, multi-sensor diagnosis offers a broader and more reliable description of machine dynamics by collecting vibration responses from different locations or directions [11,12,13,14,15,29,30]. In milling machines, vibration transmission is strongly influenced by structural coupling, signal attenuation, and spatial propagation paths [31]. As a result, the same fault may be expressed differently at different sensing positions, while some weak fault signatures may only be observable from specific channels. Multi-sensor acquisition can therefore improve fault observability by capturing complementary information that is unavailable in any individual signal channel.

Data-level fusion preserves raw information but usually requires stronger synchronization and higher computational cost. Decision-level fusion is easy to implement, yet it may lose important cross-channel interactions because each sensor is processed independently before final aggregation [12,13,14,15]. Feature-level fusion provides an effective compromise between these two extremes: it preserves informative representations extracted from multiple sensors while maintaining manageable computational complexity [32,33]. This makes it particularly suitable for industrial diagnosis tasks where both accuracy and deployability are important.

### 1.3. Research Gap and Objectives

Based on the above observations, this study focuses on a practical diagnosis problem for high-precision milling machines: how to achieve accurate, robust, and computationally efficient fault recognition under noisy industrial conditions by exploiting complementary information from multiple vibration sensors. In this context, three research questions are addressed:

RQ1: To what extent does multi-directional vibration feature fusion improve diagnostic performance over single-direction monitoring, and what additional sensing and computational burden is introduced by this improvement?

RQ2: Which classifier backbone provides the most appropriate trade-off between diagnostic accuracy and computational efficiency for deployment-oriented diagnosis?

RQ3: Which fusion mechanism exhibits the strongest robustness under progressively degraded signal conditions?

These research questions directly determine the design of the subsequent experiments. Specifically, Section 4.1 quantifies both the diagnostic benefit and the additional processing burden associated with multi-directional feature fusion relative to single-direction monitoring. Section 4.2 analyzes the accuracy–complexity trade-off among different classifier backbones, and Section 4.3 evaluates and quantitatively compares the robustness of different fusion mechanisms under progressively degraded signal conditions.

## 2. Materials and Methods

### 2.1. Framework Overview

**Figure 1:**
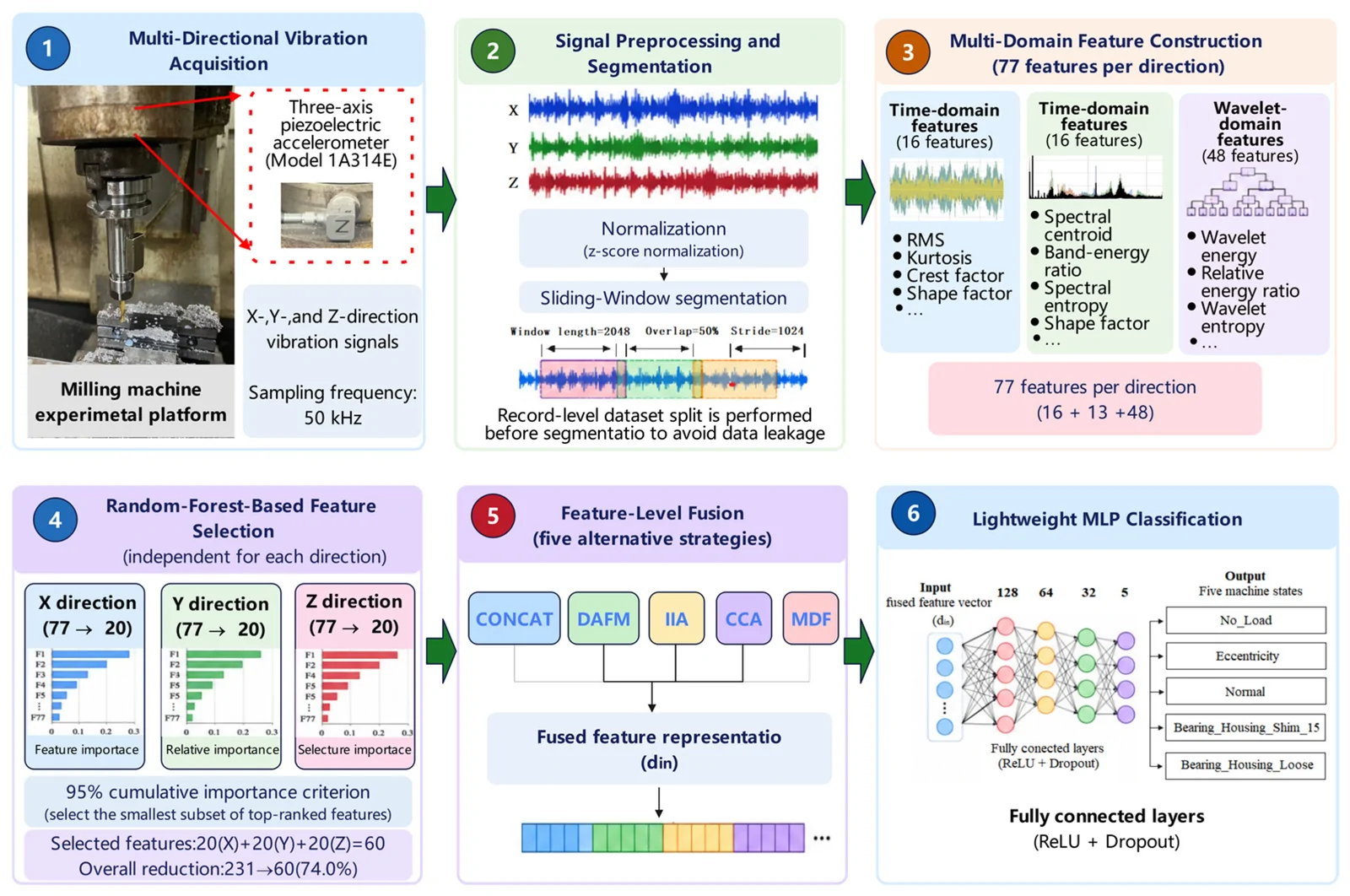
Overall framework of the proposed multi-sensor feature-level fusion method for milling-machine state diagnosis.

The overall framework of the proposed method is illustrated in Figure 1. The objective is to achieve accurate and deployment-oriented machine-state diagnosis for high-precision milling machines by integrating complementary vibration information from three sensing directions within a compact feature-level fusion pipeline. The framework consists of six main stages: three-direction vibration acquisition, signal preprocessing and segmentation, multi-domain feature construction, random-forest-based feature selection, feature-level fusion, and lightweight classification.

First, the X-, Y-, and Z-direction vibration signals are simultaneously acquired using a single three-axis piezoelectric accelerometer at a sampling frequency of 50 kHz. Second, the signals are normalized and segmented using a sliding window of 2048 points with 50% overlap. Third, 77 handcrafted features are constructed for each sensing direction from the time, frequency, and wavelet domains. Fourth, random-forest-based feature selection is independently performed for the three directions, retaining 20 features from each direction according to a 95% cumulative importance criterion. Fifth, the selected features are integrated using one of five alternative feature-level fusion mechanisms: CONCAT, DAFM, IIA, CCA, or MDF. Finally, the fused representation is fed into a lightweight MLP classifier for five-class machine-state recognition.

This framework is designed to exploit complementary vibration information from different sensing directions while controlling feature redundancy and downstream computational complexity. Performing feature selection independently for each sensing direction also helps preserve diagnostically informative information from all three directions before fusion. In addition, the dataset is partitioned at the raw acquisition-record level before overlapping window segmentation, as detailed in Section 3.1, thereby preventing segments originating from the same record from being shared among the training, validation, and test subsets.

### 2.2. Signal Preprocessing and Multi-Domain Feature Construction

Let the multi-sensor vibration signals collected from the milling machine be denoted as x(m)(t), where m indicates the sensor channel. In this study, signals from three sensing directions are considered. To reduce scale inconsistency among channels and improve training stability, each raw signal is first normalized. After normalization, a sliding-window segmentation strategy is adopted to divide continuous signals into fixed-length samples. The window length is set to 2048 points with 50% overlap, which allows each sample to preserve sufficient local fault information while increasing the number of usable training instances.

For each segmented sample, handcrafted features are extracted independently from each sensing direction in the time, frequency, and wavelet domains. In total, 77 features are constructed for each vibration channel, comprising 16 time-domain features, 13 frequency-domain features, and 48 wavelet-domain features. Consequently, each sensing direction produces a 77-dimensional multi-domain feature vector, and the three sensing directions together form an initial 231-dimensional feature pool.

The 16 time-domain features include the mean, standard deviation, variance, root mean square (RMS), maximum value, minimum value, peak-to-peak value, absolute peak value, mean absolute value, root amplitude, skewness, kurtosis, crest factor, impulse factor, shape factor, and clearance factor. These indicators characterize the overall amplitude level, signal dispersion, waveform shape, and impulsive characteristics of the vibration response.

For frequency-domain analysis, the fast Fourier transform (FFT) is first applied to each segmented signal. The 13 frequency-domain features consist of eight global spectral indicators—spectral centroid, median frequency, root mean square frequency (RMSF), spectral spread, dominant frequency, spectral peak amplitude, spectral energy, and spectral entropy—together with five band-energy ratios calculated over 0–0.5 kHz, 0.5–1 kHz, 1–2 kHz, 2–5 kHz, and 5–10 kHz. These features characterize the location and dispersion of spectral energy, dominant spectral components, and fault-related energy redistribution.

For the wavelet-domain representation, a Daubechies-4 (db4) wavelet is used as the mother wavelet, and a four-level wavelet packet decomposition (WPD) is performed. Unlike conventional discrete wavelet decomposition, WPD decomposes both approximation and detail components and therefore provides a more refined representation of localized frequency information [34]. At the fourth decomposition level, the signal is divided into 16 terminal subbands. For each terminal subband, three descriptors—wavelet energy, relative energy ratio, and wavelet entropy—are calculated, resulting in 48 wavelet-domain features (16 subbands × 3 descriptors).

With a sampling frequency of 50 kHz, the corresponding Nyquist frequency is 25 kHz. Therefore, the bandwidth of each terminal subband at the fourth WPD level is approximately 25/16 = 1.5625 kHz. The four-level decomposition provides a sufficiently refined representation of localized spectral-energy variations while maintaining a manageable feature dimensionality.

Consequently, each sensing direction produces a 77-dimensional multi-domain feature vector, yielding a total of 231 handcrafted features across the three sensing directions. The three 77-dimensional channel-specific feature sets are retained separately so that random-forest-based feature selection can be performed independently for the X-, Y-, and Z-directions before feature-level fusion, as described in Section 2.3.

### 2.3. Random-Forest-Based Feature Selection

Although multi-domain and multi-sensor feature construction improves representational richness, it also introduces substantial redundancy. Redundant features not only increase computational burden, but may also degrade generalization performance by introducing noise and unstable correlations. To address this issue, a random-forest-based feature selection strategy is employed before feature fusion.

Random forest is well suited for this task because it can estimate feature importance from an ensemble of decision trees while modeling nonlinear relationships between features and machine states. In the present framework, feature selection is performed independently for each sensing direction using only the training data. As described in Section 2.2, each X-, Y-, and Z-direction vibration channel initially contains 77 handcrafted features.

For each sensing direction *i* ∈ {*X*,*Y*,*Z*}, a random forest classifier is trained using the corresponding 77-dimensional feature set. The importance score $I_{j}^{\left(i\right)}$ of feature *j* is calculated according to its mean contribution to impurity reduction across the ensemble of decision trees. The importance scores within each sensing direction are normalized such that

(1) $$\sum_{j=1}^{77}I_{j}^{\left(i\right)}=1,i\in \left\{X,Y,Z\right\}$$

To avoid the arbitrariness associated with a fixed absolute importance threshold, the features within each sensing direction are ranked in descending order of importance:

(2) $$I_{\left(1\right)}^{\left(i\right)}\geq I_{\left(2\right)}^{\left(i\right)}\geq \cdots \geq I_{\left(77\right)}^{\left(i\right)}$$

The number of retained features is determined using a cumulative-importance criterion:

(3) $$k_{i}=\min\left\{m:\sum_{j=1}^{m}I_{\left(j\right)}^{\left(i\right)}\geq 0.95\right\}$$

For the present dataset, the 95% cumulative-importance criterion retained 20 features from each sensing direction, reducing each original 77-dimensional channel-specific representation to 20 dimensions. Consequently, the three sensing directions jointly provide a 60-dimensional selected feature representation (3 × 20) for subsequent feature-level fusion. Overall, the feature dimensionality is reduced from 231 to 60, corresponding to an approximately 74.0% reduction.

Performing feature selection independently for the three sensing directions ensures that diagnostically informative features from all directions are preserved before fusion and prevents highly ranked features from one direction from suppressing potentially complementary information from another direction. Importantly, both random-forest fitting and feature selection are performed exclusively on the training set to prevent information leakage from the validation and test subsets.

After feature selection, the resulting channel-specific feature vectors are denoted by **F***_X_*, **F***_Y_* and **F***_Z_*, where

**F***_X_*, **F***_Y_*, **F***_Z_* ∈ ℝ^20^ (4)

These representations are subsequently used as the inputs to the feature-level fusion mechanisms described in Section 2.4.

After feature selection, the retained features constitute compact representations for the subsequent feature-level fusion mechanisms.

### 2.4. Fusion Mechanisms and Classifier Design

To exploit complementary information from multiple vibration channels, feature-level fusion is performed on the selected channel-wise representations. Rather than assuming a single fusion strategy to be universally optimal, this study considers five representative fusion mechanisms. The baseline CONCAT method directly concatenates these three channel-specific representations, as defined in (5). Although computationally efficient, this strategy does not explicitly model the relative importance or interaction of different channels.

**F**_concat_ = **F***_X_*||**F***_Y_*||**F***_Z_* (5)

where **F***_X_*, **F***_Y_*, and **F***_Z_* denote the selected 20-dimensional feature vectors from the X-, Y-, and Z-directions, respectively, and || denotes concatenation. Therefore, the CONCAT representation has a dimensionality of 60.

To provide stronger adaptive integration, three comparative attention-based mechanisms are further considered. DAFM assigns channel-wise adaptive weights according to the diagnostic relevance of each sensing direction. After random-forest-based feature selection, each sensing direction is represented by a 20-dimensional feature vector. The adaptive weighting and fusion processes are defined as

*α_i_* = *σ*(**W***_a_* ⋅ **F***_i_* + *b_a_*) (6)

(7) $$F_{\mathrm{DAFM}}=\sum_{i=1}^{N_{s}}\alpha_{i}\cdot F_{i},\;N_{s}=3$$

where **F***_i_* ∈ ℝ^20^ denotes the selected feature vector from the *i*-th sensing direction, with *i* ∈ {*X*,*Y*,*Z*}. Accordingly, the number of sensing directions is *N_s_* = 3 and the feature dimension of each direction is *d_f_* = 20. The learnable weight matrix **W***_a_* ∈ ℝ^1×20^ and scalar bias *b_a_* ∈ ℝ are used to generate the scalar adaptive weight *α_i_* ∈ ℝ, while *σ*(·) denotes the sigmoid activation function. The weighted features from the three sensing directions are subsequently aggregated according to Equation (7), yielding the fused representation **F**_DAFM_ ∈ ℝ^20^.

IIA employs a dedicated attention network to estimate feature importance across sensors; its core attention computation and weighted aggregation are given in (8) and (9).

(8) $$S=\mathrm{Reshape}(F_{\mathrm{conncat}})\in {\mathbb{R}}^{B\times N_{S}\times d_{f}}$$

(9) $$F_{\mathrm{IIA}}=\sum_{i=1}^{N_{S}}\beta_{i}S_{:,i,:}$$

where *B* denotes the batch size, *N_s_* = 3 is the number of sensing directions, and *d_f_* = 20 is the selected feature dimensionality of each direction. Accordingly, the channel-wise feature tensor used by the attention module has the dimension **S** ∈ ℝ*^B^*^×3×20^. $S_{:,i,:}\in {\mathbb{R}}^{B\times d_{f}}$ represents the feature matrix corresponding to the *i*-th sensing direction. The coefficient *β_i_* denotes the normalized importance weight estimated by the IIA attention module.

CCA models competitive relationships among sensor features through cross-attention, and its key projection and fusion operations are described in (10) and (11). These methods are included as representative comparison strategies for evaluating different fusion paradigms.

**Q** = **SW**_Q_, **K** = **SW**_K_, **V** = **SW**_V_ (10)

(11) $$A=\mathrm{Softmax}\left(QK^{T}/\sqrt{d_{k}}\right),\;H_{\mathrm{CCA}}=\mathrm{AV}$$

where **W**_Q_, $W_{K}\in {\mathbb{R}}^{20\times d_{k}}$, and $W_{V}\in {\mathbb{R}}^{20\times d_{v}}$ are the learnable query, key, and value projection matrices, respectively. The resulting query and key tensors satisfy **Q**, $K\in {\mathbb{R}}^{B\times N_{s}\times d_{k}}$, while $V\in {\mathbb{R}}^{B\times N_{s}\times d_{v}}$. The attention matrix $A\in {\mathbb{R}}^{B\times N_{s}\times d_{s}}$ describes the pairwise competitive relationships among the three sensing directions. The sensor dimension of **H**_CCA_ is subsequently aggregated to obtain the fused representation **F**_CCA_.

Because the subsequent experiments identify MDF as the most robust fusion mechanism, its implementation is described in greater detail. Unlike the sensing-direction fusion methods described above, MDF explicitly reorganizes the retained features according to their original feature domains and performs adaptive fusion among the time-, frequency-, and wavelet-domain representations.

Let $X_{\mathrm{time}}\in {\mathbb{R}}^{d_{\mathrm{time}}},\;X_{\mathrm{freq}}\in {\mathbb{R}}^{d_{\mathrm{freq}}}$ and $X_{\mathrm{wavelet}}\in {\mathbb{R}}^{d_{\mathrm{wavelet}}}$ denote the retained feature vectors belonging to the time, frequency, and wavelet domains after random-forest-based feature selection, with dimensions

*d*_time_ + *d*_freq_ + *d*_wavelet_ = 60

To maintain a lightweight architecture while enabling direct cross-domain fusion, the three domain branches employ the same encoder structure. Each domain encoder consists of only one fully connected projection layer with 32 output neurons followed by a ReLU activation function, without additional hidden layers. The three domain-specific feature vectors are therefore mapped into a common 32-dimensional latent space as

**F**_time_ = ReLU(**W**_time_**X**_time_ + **b**_time_) (12)

**F**_freq_ = ReLU(**W**_freq_**X**_freq_ + **b**_freq_) (13)

**F**_wavelet_ = ReLU(**W**_wavelet_**X**_wavelet_ + **b**_wavelet_) (14)

where for domain *d* = {time,freq,wavelet}, $W_{d}\in {\mathbb{R}}^{32\times d_{d}}$ and **b***_d_* ∈ ℝ^32^ denote the learnable projection matrix and bias vector, respectively, and **F***_d_* ∈ ℝ^32^ denotes the resulting domain-specific representation.

To adaptively determine the relative contribution of the three feature domains, a learnable attention vector **w***_d_* ∈ ℝ^32^ is introduced for each domain. The corresponding domain-attention weights are normalized through a softmax operation:

(15) $$\alpha_{d}=\exp(w_{d}^{T}F_{d})/\sum_{j\in d}\exp(w_{j}^{T}F_{j})$$

where *α_d_* denotes the normalized attention weight of domain *d* and satisfies

$$\sum_{d\in D}\alpha_{d}=1$$

Finally, the MDF representation is obtained through an attention-weighted sum of the three domain representations:

**F**_MDF_ = *α*_time_**F**_time_ + *α*_freq_**F**_freq_ + *α*_wavelet_**F**_wavelet_ (16)

yielding a 32-dimensional fused representation, i.e., **F**_MDF_ ∈ ℝ^32^.

The lightweight nature of MDF is further reflected in its compact parameterization. Because the total dimensionality of the retained features across the three domains is 60, the three domain-specific projection layers contain

32(*d*_time_ + *d*_freq_ + *d*_wavelet_) + 3 × 32 = 32 × 60 + 96 = 2016

trainable parameters. The three 32-dimensional attention vectors introduce an additional

3 × 32 = 96

parameters. Therefore, the complete MDF module contains 2112 trainable parameters, corresponding to approximately 2.11 K parameters. The MDF module does not introduce convolutional layers, recurrent structures, multi-head attention, or deep domain-specific subnetworks. All projection and attention parameters are jointly optimized with the downstream classifier through backpropagation using the training configuration described in Section 3.2.

After feature fusion, the resulting representation is fed into a lightweight MLP classifier. The MLP is selected as the default classifier because the input representation has already undergone structured signal preprocessing, multi-domain feature construction, supervised feature selection, and feature-level fusion. Under this setting, a compact fully connected architecture is sufficient to model the remaining nonlinear decision boundaries without introducing excessive computational cost. The classifier architecture is described below, while the detailed optimization and training configuration is provided in Section 3.2. Additional CNN-, MLP-Mixer-, and Transformer-based backbones are evaluated only as comparison models [35,36,37,38].

The default lightweight MLP classifier consists of three fully connected hidden layers containing 128, 64, and 32 neurons, respectively, followed by a five-neuron output layer corresponding to the five machine states. Each hidden layer follows the sequence of linear transformation, batch normalization, ReLU activation, and dropout, with the dropout probability fixed at 0.3. The classifier architecture can therefore be expressed as

*d*_in_ → 128 → 64 → 32 → 5

where *d*_in_ denotes the dimensionality of the fused representation produced by the corresponding fusion mechanism.

Including the trainable affine parameters of the batch-normalization layers, the number of trainable parameters of the MLP classifier is

$$\begin{array}{ll} P_{\mathrm{MLP}}= & (128d_{\mathrm{in}}+128)+2(128)+(128\times 64+64)+2(64)\;\\ & +(64\times 32+32)+2(32)+(32\times 5+5) \end{array}$$

which simplifies to

*P*_MLP_ = 128*d*_in_ + 11077

For a 60-dimensional input representation, such as the direct CONCAT representation, the classifier contains

*P*_MLP_ = 18,757

trainable parameters, corresponding to approximately 18.76 K parameters. In contrast, because MDF produces a 32-dimensional fused representation, the corresponding MLP classifier contains

*P*_MLP_ = 15,173

trainable parameters, corresponding to approximately 15.17 K parameters.

Considering the fully connected layers and counting one multiply-add operation as two floating-point operations, the main forward-pass computation of the classifier is

$${\mathrm{FLOP}}_{s_{\mathrm{MLP}}}=2(128d_{\mathrm{in}}+128\times 64+64\times 32+32\times 5)$$

For *d*_in_ = 60, this corresponds to 36,160 FLOPs, or approximately 0.036 MFLOPs per sample. For the 32-dimensional MDF representation, the corresponding classifier computation is 28,992 FLOPs, or approximately 0.029 MFLOPs per sample. These results further demonstrate that both the MDF module and the downstream classifier maintain low model complexity, supporting deployment in resource-constrained machine-monitoring applications.

## 3. Experimental Setup

### 3.1. Platform, Dataset, and Split Protocol

**Figure 2:**
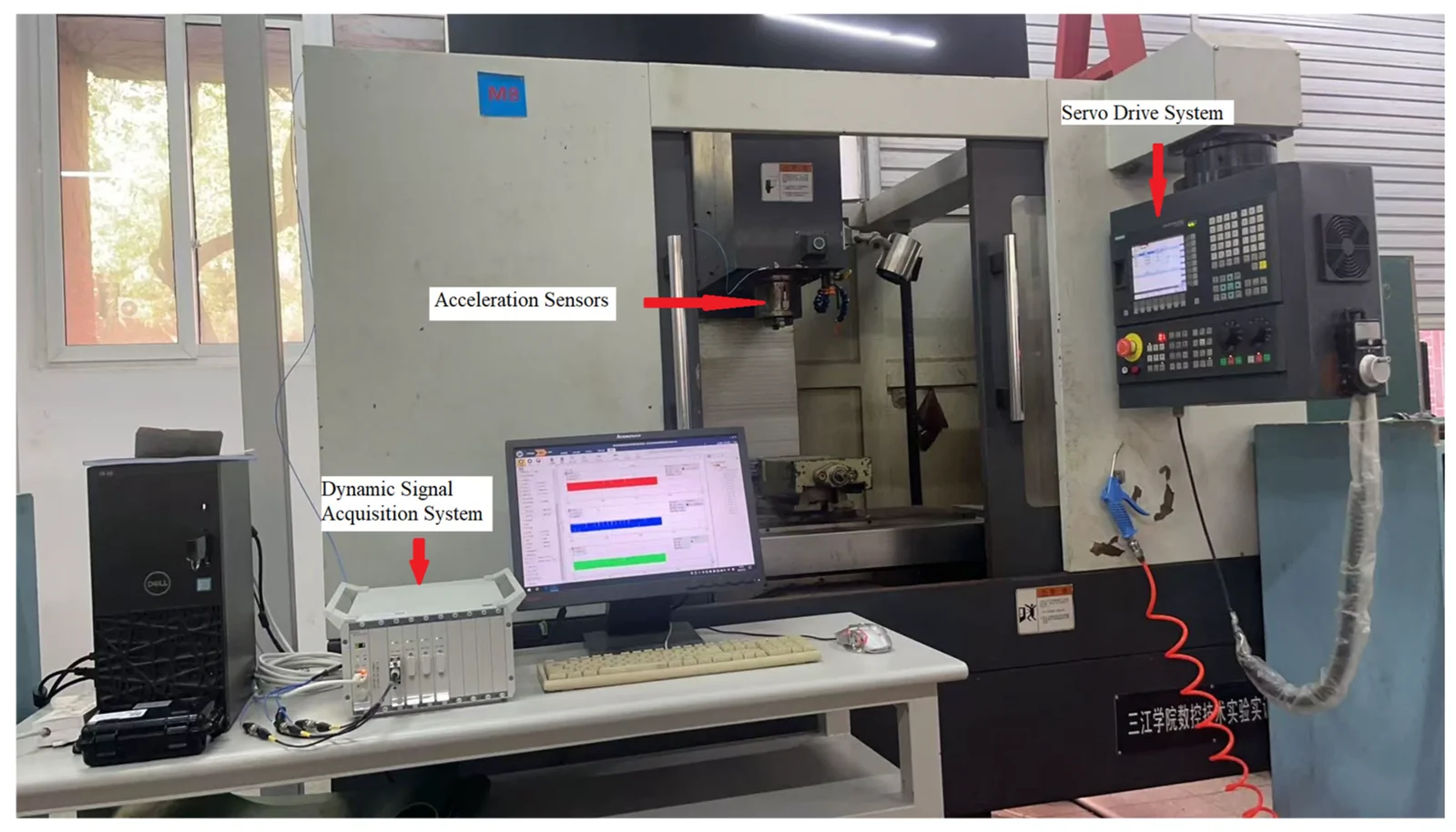
Experimental platform for milling-machine vibration acquisition.

Experiments were carried out on a CNC milling machine (model XK713CG-11,Nanjing No. 2 Machine Tool Factory, Nanjing, China), equipped with a three-axis piezoelectric accelerometer (model 1A314E, Jiangsu Donghua Testing Technology Co., Ltd., Jingjiang, China) and a dynamic signal acquisition system (model DH5922D, Jiangsu Donghua Testing Technology Co., Ltd., Jingjiang, China)). The vibration signals were sampled at 50 kHz to ensure sufficient temporal resolution for capturing the transient dynamic characteristics of the milling process. Figure 2 illustrates the experimental platform, including the machine tool, sensor installation, and data acquisition system.

Five machine states were considered in this study, namely No_Load, Eccentricity, Normal, Bearing_Housing_Shim_15, and Bearing_Housing_Loose. Among them, No_Load and Normal represent non-fault operating states, whereas Eccentricity, Bearing_Housing_Shim_15, and Bearing_Housing_Loose represent abnormal or fault-related states. For each machine state, 10 independent raw acquisition records were collected, resulting in a total of 50 raw records. Each record contains simultaneously acquired vibration signals from the X-, Y-, and Z-directions. A representative raw record contains 1,514,277 sampling points per sensing direction, corresponding to approximately 30.29 s of continuous acquisition at the sampling frequency of 50 kHz.

To avoid potential information leakage caused by overlapping window segmentation, the dataset was partitioned at the raw acquisition-record level rather than at the segmented-sample level. Specifically, for each machine state, the 10 original records were divided into 7 training records, 1 validation record, and 2 test records, corresponding to a record-level train/validation/test ratio of 70%/10%/20%. The same partitioning strategy was applied independently to all five machine states, resulting in 35 training records, 5 validation records, and 10 test records in total. Because the same number of raw records from each machine state was assigned to each subset, the class distribution is balanced at the record level.

Only after the record-level split were the signals within each subset independently segmented using a sliding window of 2048 points with 50% overlap, corresponding to a stride of 1024 points. For example, the representative record containing 1,514,277 sampling points generates 1477 segmented samples using this windowing strategy. The number of segmented samples generated from a raw record therefore depends on its original record length.

Importantly, all overlapping segments generated from the same raw acquisition record were retained exclusively within the subset to which that record was assigned. Thus, segments originating from the same continuous acquisition record were never shared among the training, validation, and test subsets. This record-level partitioning strategy prevents highly correlated overlapping windows from the same raw signal from appearing simultaneously in the training and evaluation sets, thereby avoiding potential information leakage and providing a more reliable assessment of model generalization.

Table 1 summarizes the five machine states, the number of raw acquisition records, and their record-level allocation to the training, validation, and test subsets.

### 3.2. Implementation Details and Evaluation Metrics

All models were implemented under the same experimental framework and trained using an identical optimization protocol to ensure a consistent comparison across different fusion mechanisms and classifier backbones. AdamW was adopted as the optimizer because its decoupled weight-decay mechanism provides stable optimization while allowing explicit regularization control. The initial learning rate was set to 0.001, and a weight decay of 0.01 was employed to reduce overfitting under the limited-data setting.

The batch size was fixed at 32 as a compromise between gradient stability and computational efficiency, and the maximum number of training epochs was set to 100 for all models to provide a consistent training horizon and enable direct comparison of their optimization and generalization trajectories. As described in Section 3.1, the five machine states are balanced at the raw-record level, and the dataset was partitioned at the raw acquisition-record level using a train/validation/test ratio of 70%/10%/20%. The validation subset was used to monitor model generalization and learning behavior during training. Therefore, the observed epoch-wise fluctuations cannot be attributed to class imbalance.

To further improve generalization and training stability, a dropout rate of 0.3 was employed where applicable, while label smoothing with a coefficient of 0.05 was used to alleviate overconfident predictions. Gradient clipping with a maximum *L*_2_-norm of 1.0 was applied during backpropagation to suppress excessively large gradient updates. In addition, the learning rate was adaptively adjusted using a ReduceLROnPlateau scheduler based on the validation loss. If the validation loss did not improve for eight consecutive epochs, the learning rate was reduced by a factor of 0.5, with a minimum learning rate of 1 × 10^−5^. It should be noted that ReduceLROnPlateau is an adaptive scheduler triggered by stagnation of the validation loss rather than by predetermined epoch positions. Therefore, a learning-rate reduction does not necessarily produce an immediate step-like decrease in the corresponding loss curve.

The major optimization settings, including the optimizer, initial learning rate, weight decay, batch size, maximum number of epochs, label smoothing, gradient clipping, and learning-rate scheduling strategy, were kept identical across the compared models. No model-specific optimization hyperparameter tuning was performed. Therefore, the comparison was conducted under a common training protocol to minimize the influence of inconsistent optimization settings. The detailed training configuration is summarized in Table 2.

In addition to the lightweight MLP classifier, compact CNN-, MLP-Mixer-, and Transformer-based backbones were evaluated using the same data partition and training protocol. Because these architectures have intrinsically different structural designs, exact parameter matching was not imposed. Accordingly, the comparison is interpreted as an accuracy–complexity trade-off analysis, rather than a strictly capacity-controlled architectural ablation.

Diagnostic performance was primarily evaluated using classification accuracy. Confusion matrices and feature-space visualizations were additionally used to examine inter-class separability and error distributions. For the noise-robustness experiments, the mean accuracy and standard deviation obtained under different signal-to-noise ratios were reported to quantify performance stability. Model complexity was evaluated using the number of trainable parameters and floating-point operations (FLOPs) per inference, providing an additional basis for assessing the suitability of the evaluated models for resource-constrained deployment.

For the noise-robustness experiments, each fusion mechanism was independently trained and evaluated 10 times at each SNR level, with both the Gaussian-noise realization and model initialization regenerated for every run. The resulting test accuracies are reported as mean ± standard deviation. The detailed noise-injection procedure is described in Section 3.3.

### 3.3. Noise-Injection Protocol

To provide a reproducible evaluation of noise robustness, additive white Gaussian noise (AWGN) was introduced according to six prescribed signal-to-noise ratio (SNR) levels: 0, −2, −4, −6, −8, and −10 dB. For a vibration signal **X** = [*x*_1_,*x*_2_,⋯,*x_N_*], its average signal power is calculated as

(17) $$P_{s}=\frac{1}{N}\sum_{i=1}^{N}x_{i}^{2}$$

For a target SNR value *S* in decibels, the required noise power is determined by

(18) $$P_{n}=\frac{P_{s}}{10^{S/10}}$$

Zero-mean Gaussian noise with the corresponding variance is then generated as

(19) $$n∼N(0,P_{n})$$

and the noise-contaminated vibration signal is obtained as

(20) $$\tilde{X}=X+n$$

The noise-robustness experiments followed the same record-level data partition described in Section 3.1. For each target SNR, independent AWGN realizations were generated for the training, validation, and test subsets, thereby preventing the same artificial noise realization from being shared across different subsets. The same target SNR was applied to all three subsets within a given experimental condition.

A separate model was trained from scratch for each SNR level using the corresponding noise-contaminated training data. The validation subset contaminated at the same target SNR was used for monitoring model generalization and learning behavior, and the final diagnostic performance was evaluated on the independently noise-contaminated test subset. Therefore, the results at different SNR levels represent independently trained models under matched training, validation, and testing noise conditions, rather than the evaluation of a single clean-trained model under progressively noisier test inputs.

To reduce the influence of stochastic noise realizations and random model initialization, each fusion mechanism at each SNR level was independently evaluated 10 times. For every repetition, the AWGN realizations were regenerated and the model parameters were reinitialized before training. The accuracy values reported in Table 3 are therefore expressed as the mean ± standard deviation over 10 independent runs. All fusion mechanisms were evaluated using exactly the same SNR levels, data partition, training protocol, and number of repetitions to ensure a consistent robustness comparison.

## 4. Results and Discussion

### 4.1. Benefit of Multi-Directional Feature Fusion

The first objective of the experimental analysis is to determine whether multi-directional vibration feature fusion can provide a clear diagnostic advantage over single-direction monitoring. To address this issue, the performance of single-direction models and the multi-direction fused-feature model was compared from three complementary perspectives, namely training behavior, classification performance, and feature-space separability.

**Figure 3:**
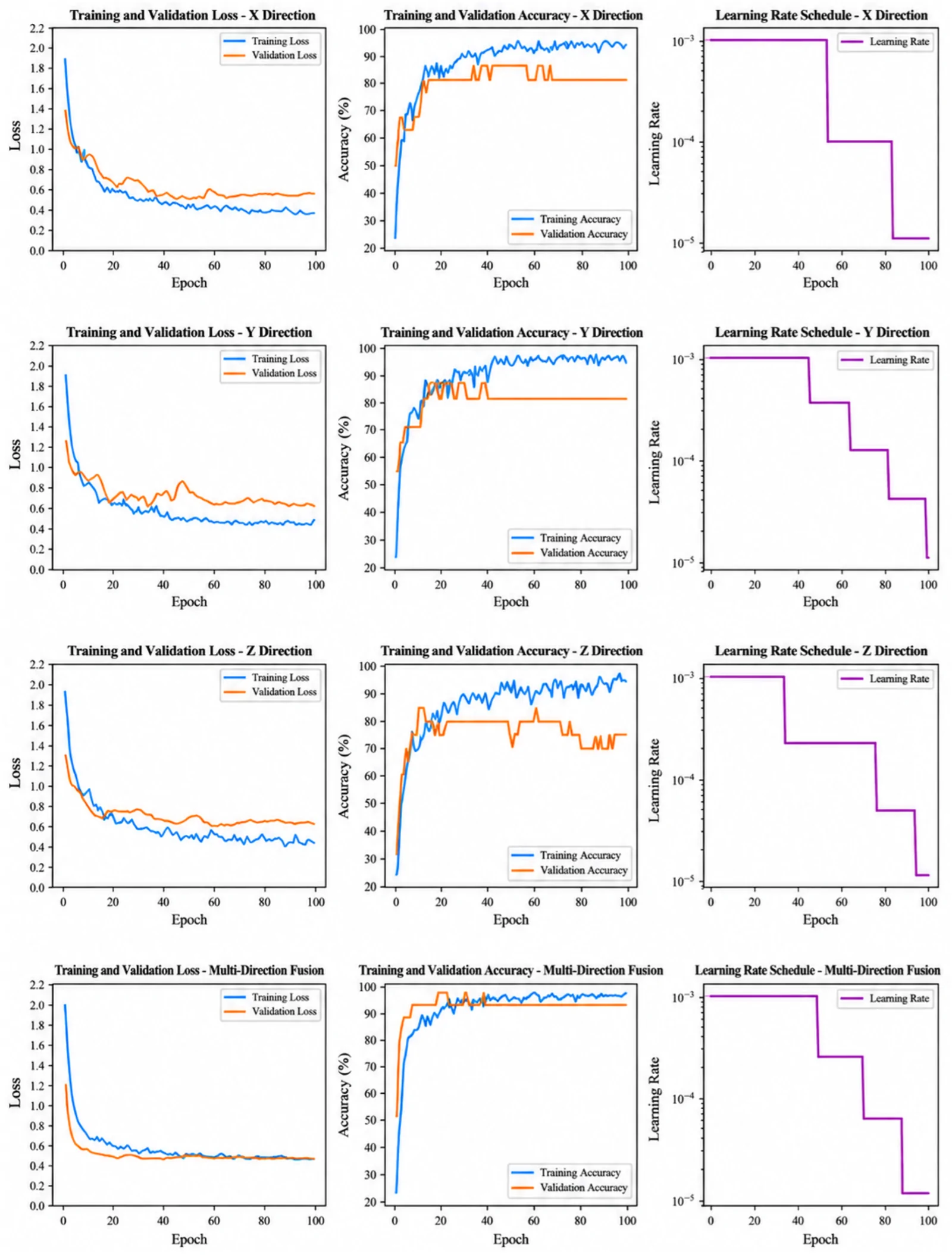
Training behavior of the X-, Y-, Z-direction and multi-direction fusion models.

As shown in Figure 3, the raw epoch-wise training trajectories exhibit noticeable stochastic fluctuations, particularly for the single-direction models. The curves are presented without smoothing in order to preserve the actual optimization behavior. Such fluctuations are expected to some extent in mini-batch stochastic optimization and, by themselves, do not necessarily indicate a failure of convergence. In addition, as described in Section 3.1, the five machine states are balanced at the raw-record level, excluding class imbalance as the main source of the observed fluctuations.

Nevertheless, the validation losses of the X-, Y-, and Z-direction models tend to reach their minima earlier than the end of the 100-epoch training period and subsequently increase to varying degrees while the corresponding training losses continue to decrease. This divergence indicates a certain degree of overfitting during the later training stage. The adopted training protocol incorporates several regularization mechanisms, including AdamW weight decay, dropout, label smoothing, and gradient clipping, to improve generalization. In comparison, the fused-feature model exhibits a smaller training–validation discrepancy and more stable validation behavior, suggesting that the complementary information provided by multi-direction fusion produces a more robust and discriminative representation for downstream classification. The learning-rate trajectories in Figure 3 are generated by the ReduceLROnPlateau scheduler, which monitors the validation loss and reduces the learning rate when no improvement is observed for eight consecutive epochs. Unlike a fixed-step learning-rate schedule, this adaptive strategy does not require an immediate discontinuous decrease in the loss after each learning-rate reduction. Instead, the reduced learning rate is intended to provide finer parameter updates when validation improvement stagnates.

To further quantify the convergence behavior rather than relying solely on visual inspection of the training curves, several convergence indicators were calculated for the four model configurations. These include the final training loss, final validation loss, the absolute training–validation loss gap, and the relative change in training loss over the last 10 epochs. The absolute loss gap is defined as

Δ*L* = |*L*_val_ − *L*_train_| (21)

and the relative training-loss change over the last 10 epochs is calculated as

(22) $$R_{10}=\frac{\left|L_{100}-L_{90}\right|}{L_{90}}\times 100\%$$

The corresponding results are summarized in Table 3.

As summarized in Table 3, the single-direction models exhibit noticeable discrepancies between the final training and validation losses, with absolute loss gaps of 0.17, 0.11, and 0.23 for the X-, Y-, and Z-direction models, respectively. In contrast, the multi-direction fused-feature model shows a substantially smaller loss gap of 0.03. The relative changes in training loss over the final 10 epochs are 7.8%, 4.3%, 5.3%, and 2.5%, respectively, with the fused-feature model exhibiting the smallest late-stage variation. These quantitative results are consistent with the trends observed in Figure 3 and indicate that multi-direction feature fusion provides more stable optimization and better generalization behavior than the individual sensing directions.

**Figure 4:**
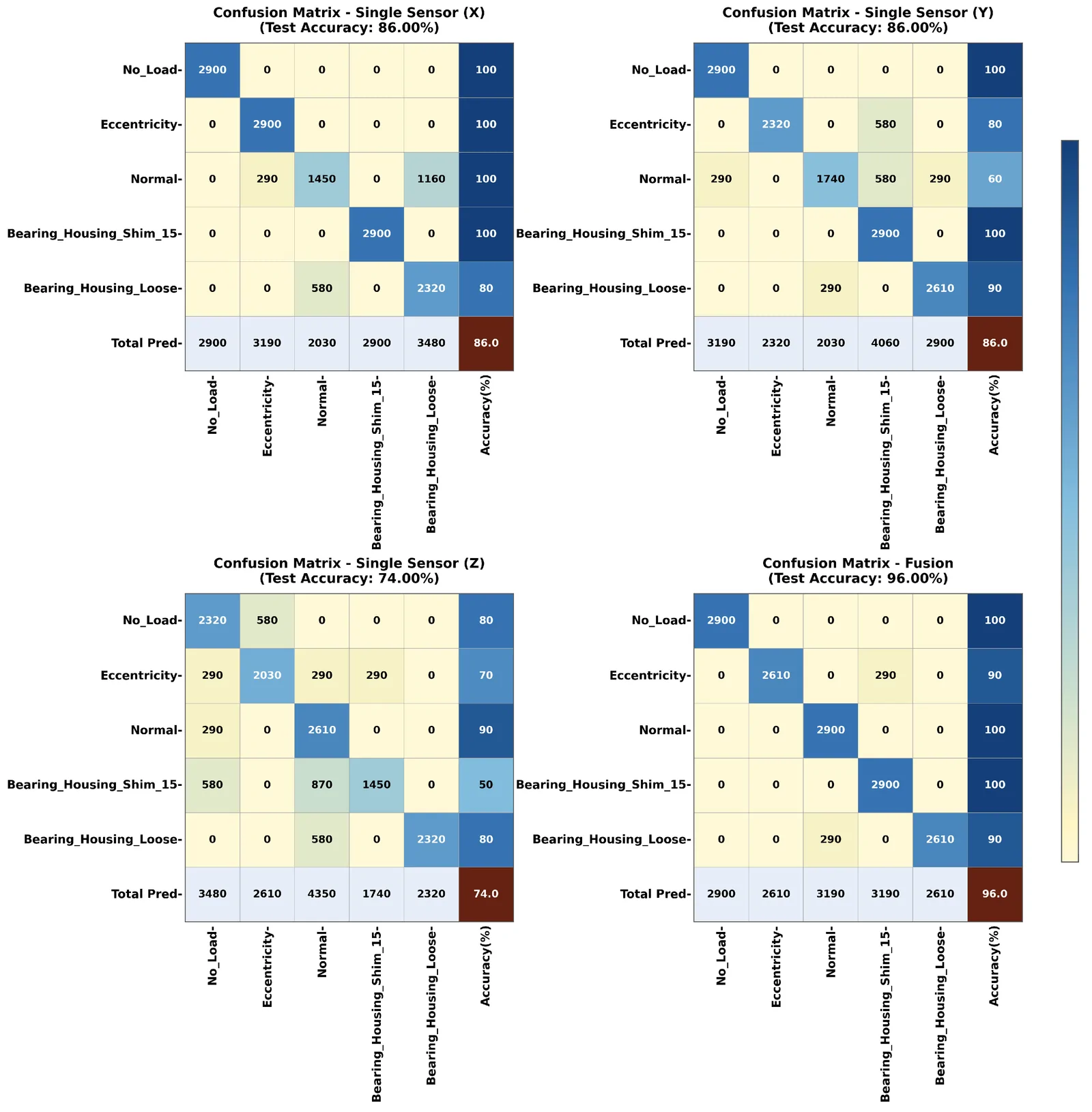
Confusion matrices of single-sensor and fused-feature models.

The final classification results are further illustrated in Figure 4. The X-direction and Y-direction single-direction models each achieve an accuracy of 86.00%, whereas the Z-direction model achieves only 74.00%. These results indicate that the diagnostic information available from an individual sensing direction is incomplete and direction-dependent. However, the lower performance of the Z-direction model does not imply that this direction is redundant. Instead, the improvement obtained after fusion demonstrates that different vibration directions contain complementary information that cannot be fully captured by any single direction alone. In contrast, the fused-feature model achieves an accuracy of 96.00%, with misclassifications limited to a small number of samples from the Eccentricity and Bearing_Housing_Loose states. This substantial improvement confirms the effectiveness of multi-direction feature fusion for machine-state recognition.

From a quantitative benefit perspective, the fused model improves diagnostic accuracy by 10 percentage points compared with the best single-direction model (86.00% to 96.00%) and by 14 percentage points compared with the average single-direction accuracy (82.00% to 96.00%). Furthermore, the classification error decreases from 14.00% for the best single-direction model to 4.00% after fusion, corresponding to an approximately 71.4% relative reduction in error. These results demonstrate that the proposed fusion strategy achieves a substantial diagnostic improvement by exploiting complementary vibration information from multiple sensing directions.

From a computational-cost perspective, the improvement is achieved mainly through increased feature representation rather than additional sensing hardware or acquisition time. In the experimental platform, the X-, Y-, and Z-direction vibration signals are acquired simultaneously using a single three-axis piezoelectric accelerometer. Each sensing direction initially produces 77 handcrafted features, including time-domain, frequency-domain, and wavelet-domain descriptors, resulting in an initial 231-dimensional feature pool. Subsequently, random-forest-based feature selection is independently performed for each direction, retaining 20 informative features per direction according to the 95% cumulative importance criterion. Therefore, the final selected representation contains 60 features before fusion, reducing the original feature dimensionality by approximately 74.0% while preserving the dominant diagnostic information. This feature-selection strategy effectively limits the additional computational burden introduced by multi-direction feature fusion.

**Figure 5:**
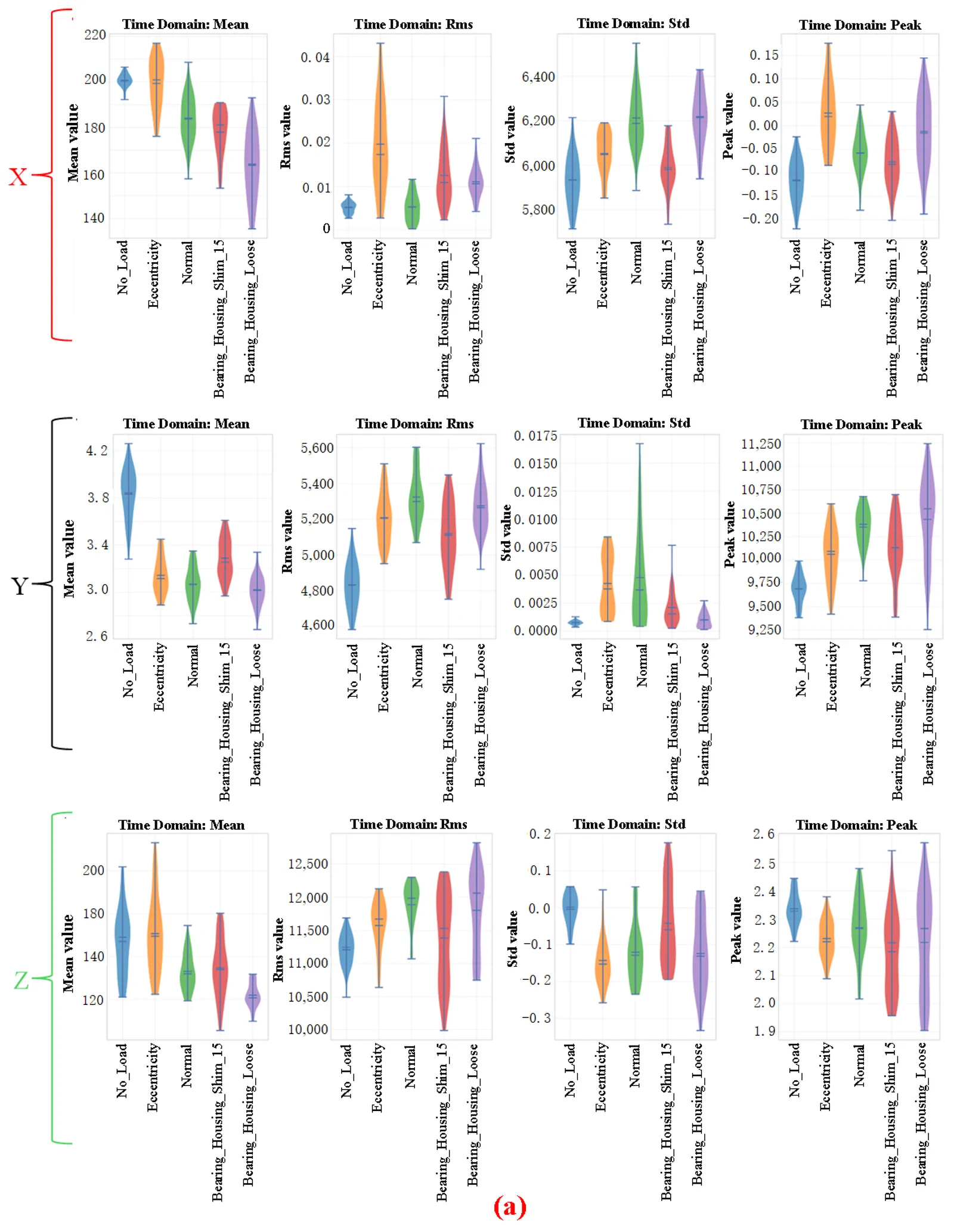

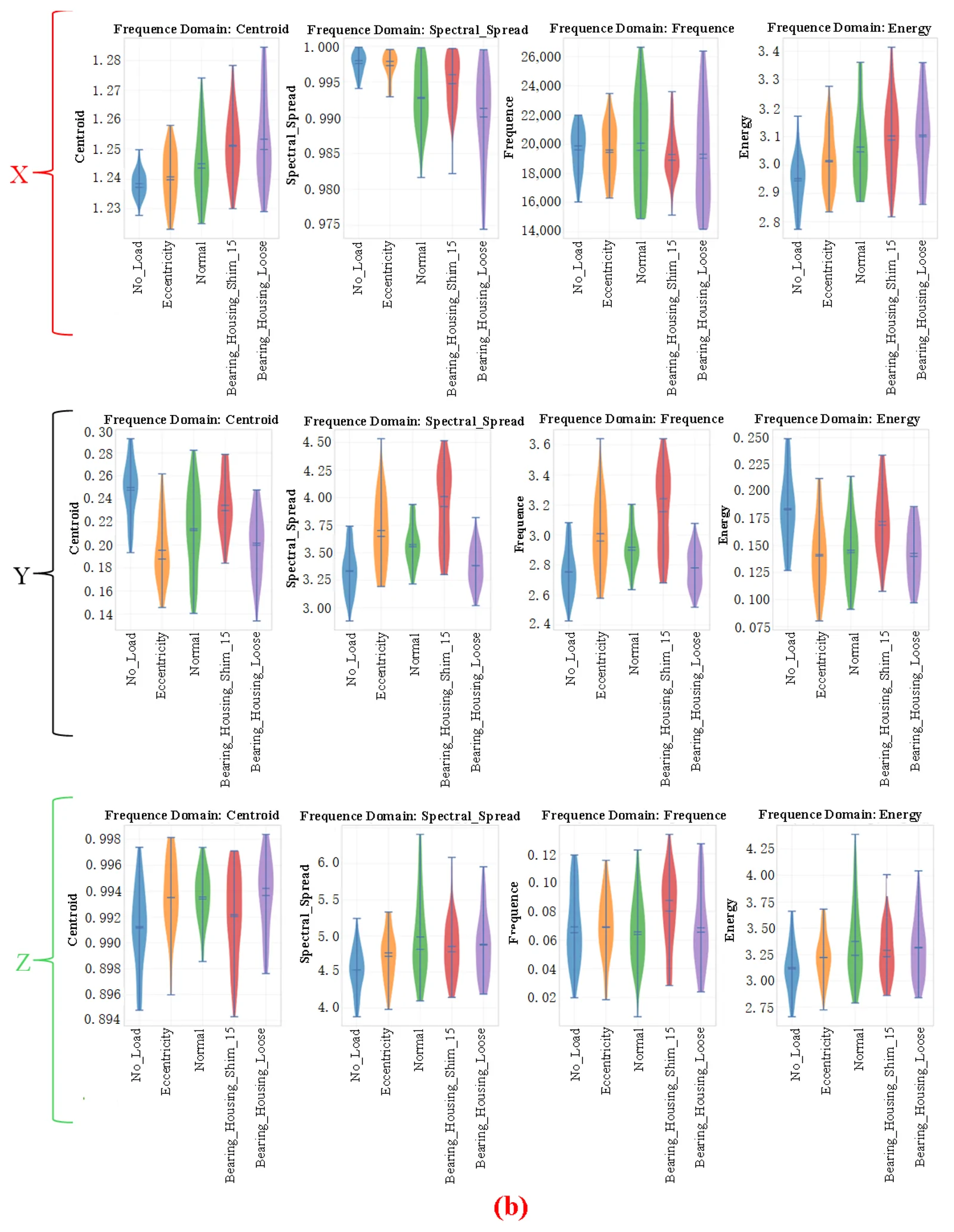
Distributions of representative features extracted from the X-, Y-, and Z-direction vibration signals: (**a**) representative time-domain features; (**b**) representative frequency-domain features.

To further explain why the fused model performs better, Figure 5 compares the feature distributions obtained from individual sensing directions and from the fused representation. It can be observed that the single-direction features preserve only partial characteristics of different machine states, and several classes still exhibit noticeable overlap. In contrast, the fused representation presents a more compact and better-structured distribution, indicating that integrating selected multi-domain features from multiple directions improves the completeness of state characterization and enhances inter-class separability.

**Figure 6:**
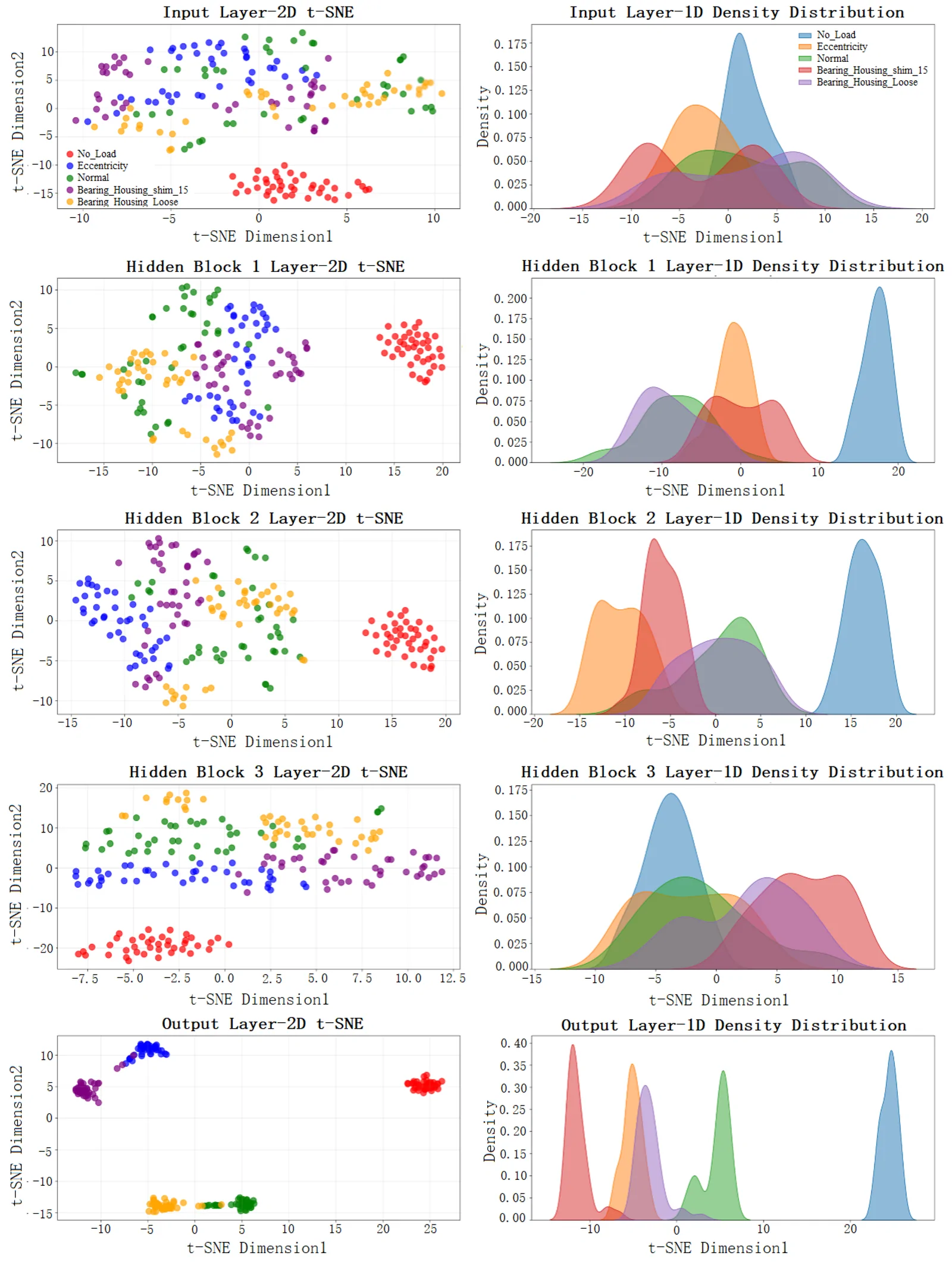
Progressive evolution of MLP feature clusters across layers.

The representational advantage of multi-direction fusion is further confirmed in Figure 6, which illustrates the progressive evolution of feature clusters across different layers of the MLP. As the fused features propagate through the network, samples belonging to the same machine state become increasingly compact, while the separation between different states becomes more evident. This phenomenon indicates that the proposed framework provides a more informative input representation that can be effectively transformed into discriminative latent features by a lightweight classifier.

Overall, the results from Figure 3, Figure 4, Figure 5 and Figure 6 and Table 3 show that multi-direction feature fusion improves feature separability and final classification performance compared with single-direction monitoring, while also exhibiting a smaller training–validation loss gap and lower late-stage training-loss variation. These findings provide a positive answer to RQ1, confirming that vibration information from different sensing directions is complementary rather than redundant.

The representational advantage of multi-sensor fusion is further confirmed in Figure 6, which shows the progressive evolution of feature clusters across different layers of the MLP. As the fused features propagate through the network, the class distributions become increasingly compact within each category and more separated across different categories. This phenomenon indicates that the proposed framework provides a feature space that is not only more informative at the input level, but also more suitable for hierarchical nonlinear discrimination by a lightweight classifier.

Taken together, the evidence from Figure 3, Figure 4, Figure 5 and Figure 6 provides a consistent answer to RQ1: multi-sensor feature fusion substantially improves optimization stability, feature separability, and final diagnostic accuracy compared with single-sensor monitoring. These results confirm that the information distributed across different sensing directions is complementary rather than redundant, thereby establishing the representational basis for the subsequent comparison of classifier backbones and fusion mechanisms.

### 4.2. Efficiency-Performance Trade-Off Across Classifier Backbones

Following the verification of the effectiveness of multi-direction feature fusion, the next question is whether the fused feature representation requires a highly complex classifier to achieve strong diagnostic performance. To investigate this issue, four representative compact classifier backbones, namely MLP, CNN, MLP-Mixer, and Transformer, were evaluated using the same fused-feature input, data partition, and training protocol. Because these models are based on substantially different architectural principles, exact parameter matching was not imposed. Instead, each backbone was configured in a compact form suitable for the present low-dimensional feature-classification task. Accordingly, the comparison focuses on the joint consideration of classification accuracy and computational complexity rather than on accuracy alone.

The classification results show that CNN achieves the highest test accuracy of 98.00%, whereas the lightweight MLP reaches 96.00%, only 2.00 percentage points lower. Although the MLP-Mixer and Transformer incorporate more sophisticated feature-interaction mechanisms, their diagnostic performance does not improve proportionally with the increase in architectural complexity. This observation indicates that, after multi-domain feature extraction, random-forest-based feature selection, and multi-direction feature fusion, the resulting representation already contains strong state-discriminative information. Consequently, increasing the complexity of the downstream classifier provides only limited additional benefit for the present task.

**Figure 7:**
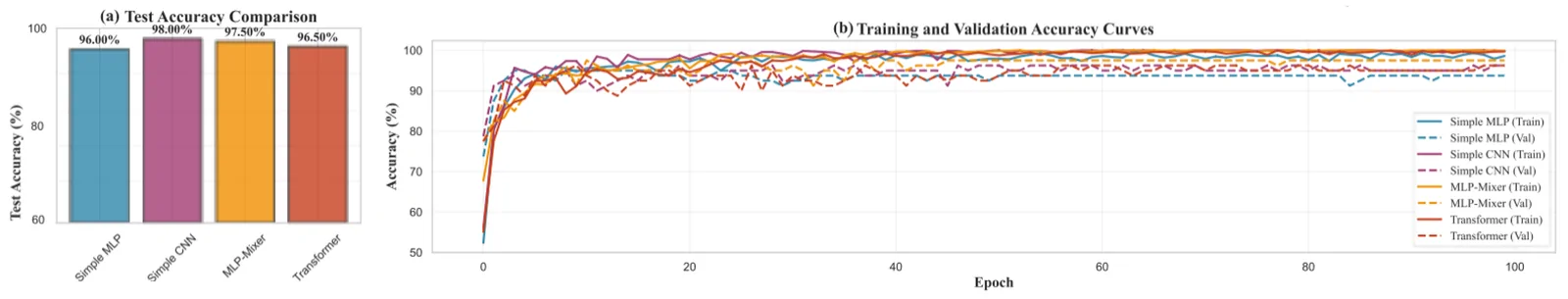
Training behavior of the evaluated classifier backbones: (**a**) Test Accuracy; (**b**) training and validation accuracy.

Figure 7 further compares the training behavior of the four classifier backbones under the common optimization protocol. Figure 7a presents the corresponding loss trajectories, whereas Figure 7b shows the accuracy curves. To make the relatively small performance differences among the evaluated backbones more clearly visible, the vertical axis of Figure 7b is restricted to the range of 60–100%. The curves show that all four models can converge under the adopted training configuration, but their convergence behavior and final generalization performance differ to some extent. In particular, the lightweight MLP maintains stable optimization behavior while achieving a test accuracy close to that of CNN, supporting its suitability as a compact downstream classifier.

**Figure 8:**
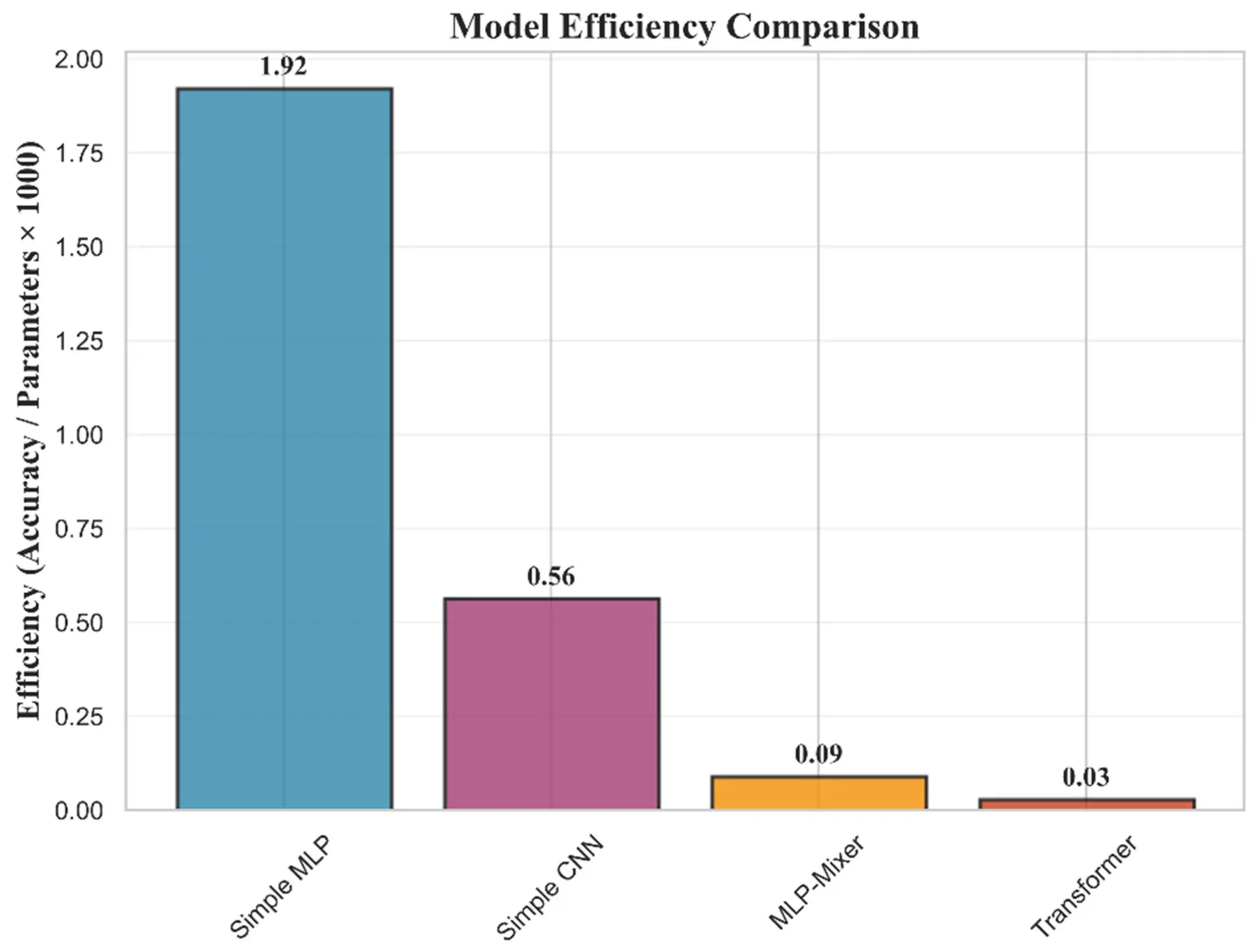
Parameter-efficiency comparison of the evaluated classifier backbones.

The computational-complexity perspective is further examined in Figure 8, which compares the evaluated classifier backbones in terms of trainable parameter count, computational cost, and parameter efficiency. When classification accuracy is considered jointly with model complexity, the lightweight MLP provides the most favorable efficiency–performance trade-off among the evaluated backbones. Although CNN improves the test accuracy from 96.00% to 98.00%, this gain amounts to only 2.00 percentage points and is accompanied by a substantially higher computational burden. Likewise, the MLP-Mixer and Transformer introduce additional structural complexity without providing a corresponding improvement in classification performance. These results indicate that maximizing network complexity is not necessary for the current feature-level diagnosis task.

The favorable performance of the lightweight MLP can be attributed to the characteristics of the input representation. Before classification, the raw vibration signals have already undergone normalization, multi-domain feature construction, supervised feature selection, and multi-direction fusion. These preceding stages substantially reduce redundant information while retaining diagnostically relevant features. Therefore, the downstream classifier is required primarily to model the nonlinear decision boundaries among the five machine states rather than to learn fault-sensitive representations directly from raw signals. Under this condition, a compact fully connected architecture is sufficient to achieve competitive diagnostic performance with a relatively low computational burden.

It should be noted that the backbone comparison is intended as an accuracy–complexity trade-off analysis rather than a strictly capacity-controlled architectural ablation. Because CNN, MLP-Mixer, Transformer, and MLP have intrinsically different computational structures, enforcing identical parameter counts would require substantial architectural modification and could compromise their representative characteristics. Therefore, all models were evaluated using the same input representation, dataset partition, optimization settings, and evaluation procedure, while their natural differences in parameter count and computational cost were explicitly retained as part of the comparison. This protocol provides a more deployment-oriented assessment of the practical cost associated with each classifier architecture.

Accordingly, the results of Figure 7 and Figure 8 provide a clear answer to RQ2: although CNN achieves the highest classification accuracy among the evaluated backbones, the lightweight MLP offers the most favorable balance between diagnostic performance and computational efficiency. It is therefore selected as the default classifier in the proposed framework for deployment-oriented machine-state diagnosis. This conclusion also provides the basis for the subsequent evaluation of different fusion mechanisms under progressively degraded signal conditions.

### 4.3. Noise Robustness and Comparison of Fusion Mechanisms

After establishing the effectiveness of multi-direction feature fusion and the deployment suitability of the lightweight MLP classifier, the final objective is to evaluate how different fusion mechanisms behave under progressively degraded signal conditions. In practical milling environments, vibration measurements are often affected by background vibration, structural interference, and measurement noise. Therefore, diagnostic robustness under low-SNR conditions is an important consideration for practical deployment. To this end, five fusion strategies, namely CONCAT, DAFM, IIA, CCA, and MDF, were evaluated at six SNR levels ranging from 0 to −10 dB.

**Figure 9:**
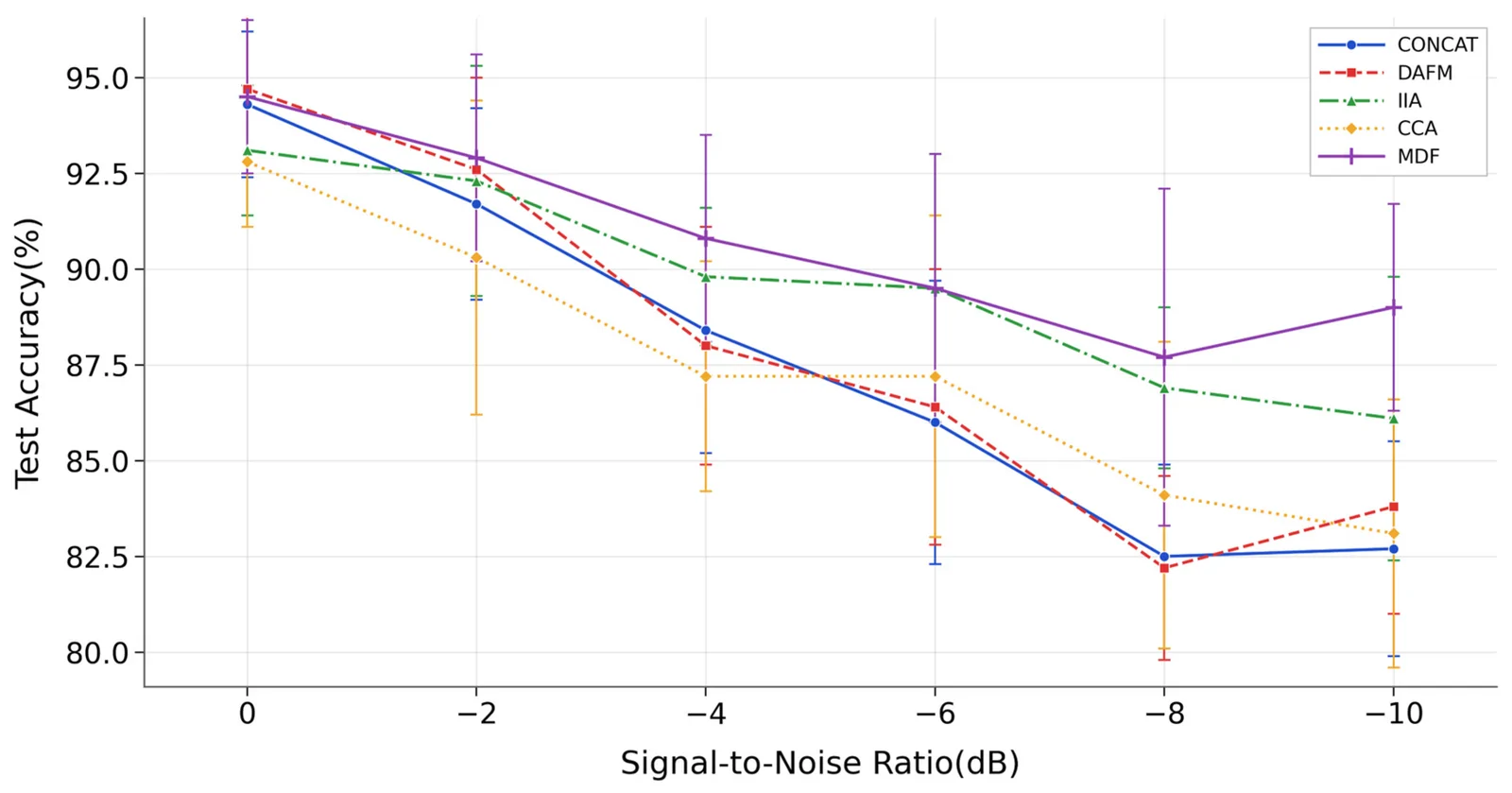
Diagnostic accuracy and linear fitted trends of different fusion mechanisms under varying SNR levels. Markers indicate the experimentally obtained mean accuracies, while the fitted lines summarize the overall degradation trends.

The overall robustness trends are summarized in Figure 9, which illustrates the variation in diagnostic accuracy as the SNR decreases. To quantify the degradation tendency of each fusion mechanism, a linear least-squares fitting was additionally applied to the mean accuracy values obtained at the six SNR levels. For ease of interpretation, the noise degradation level is defined as

*D* = SNR (23)

where *D* = 0 corresponds to 0 dB SNR and *D* = 10 corresponds to −10 dB SNR. The fitted relationship is expressed as

*A*(*D*) = *b* − *kD* (24)

where *A*(*D*) denotes the diagnostic accuracy, *b* is the fitted intercept, and *k* represents the average accuracy degradation rate in percentage points per dB. A smaller value of *k* therefore indicates stronger robustness to increasing noise interference.

As shown in Figure 9, all five fusion mechanisms exhibit an overall decline in diagnostic performance as the noise level increases, although the degradation rates differ substantially. The fitted degradation rates for CONCAT, DAFM, IIA, CCA, and MDF are 1.237, 1.243, 0.739, 0.954, and 0.636 percentage points/dB, respectively. MDF exhibits the smallest fitted degradation rate, followed by IIA, indicating that these two mechanisms are less sensitive to progressive SNR reduction. In contrast, CONCAT and DAFM show noticeably steeper fitted trends, corresponding to faster performance degradation under increasing noise.

The quantitative accuracy results are reported in Table 4. At 0 dB SNR, the five fusion mechanisms achieve comparable accuracies, ranging from 92.75% to 94.50%. As the SNR decreases, however, the differences among the methods become increasingly apparent. At −10 dB SNR, MDF still maintains an accuracy of 88.88 ± 2.53%, whereas CONCAT, DAFM, IIA, and CCA achieve 82.75 ± 3.10%,83.75 ± 2.09%,86.12 ± 3.64%, and 83.12 ± 3.59%, respectively. From 0 to −10 dB, the absolute accuracy decrease in MDF is only 5.50 percentage points, compared with 11.37, 10.75, 7.00, and 9.63 percentage points for CONCAT, DAFM, IIA, and CCA, respectively. These results demonstrate that MDF preserves diagnostic performance more effectively as the signal quality deteriorates.

The fitted degradation characteristics are further summarized in Table 5. MDF achieves the smallest degradation rate of 0.636 percentage points/dB, which is approximately 48.6% lower than that of the CONCAT baseline (1.237 percentage points/dB). IIA also exhibits relatively strong noise robustness with a degradation rate of 0.739 percentage points/dB. By contrast, DAFM produces the largest fitted degradation rate of 1.243 percentage points/dB. The corresponding *R*^2^ values indicate that the linear model provides a reasonable summary of the overall degradation tendency for all five methods. It should be noted that the linear fits are intended to characterize the average trend rather than imply an exact deterministic relationship between SNR and diagnostic accuracy. Small non-monotonic variations at individual SNR levels can arise from statistical fluctuations across repeated experiments.

**Figure 10:**
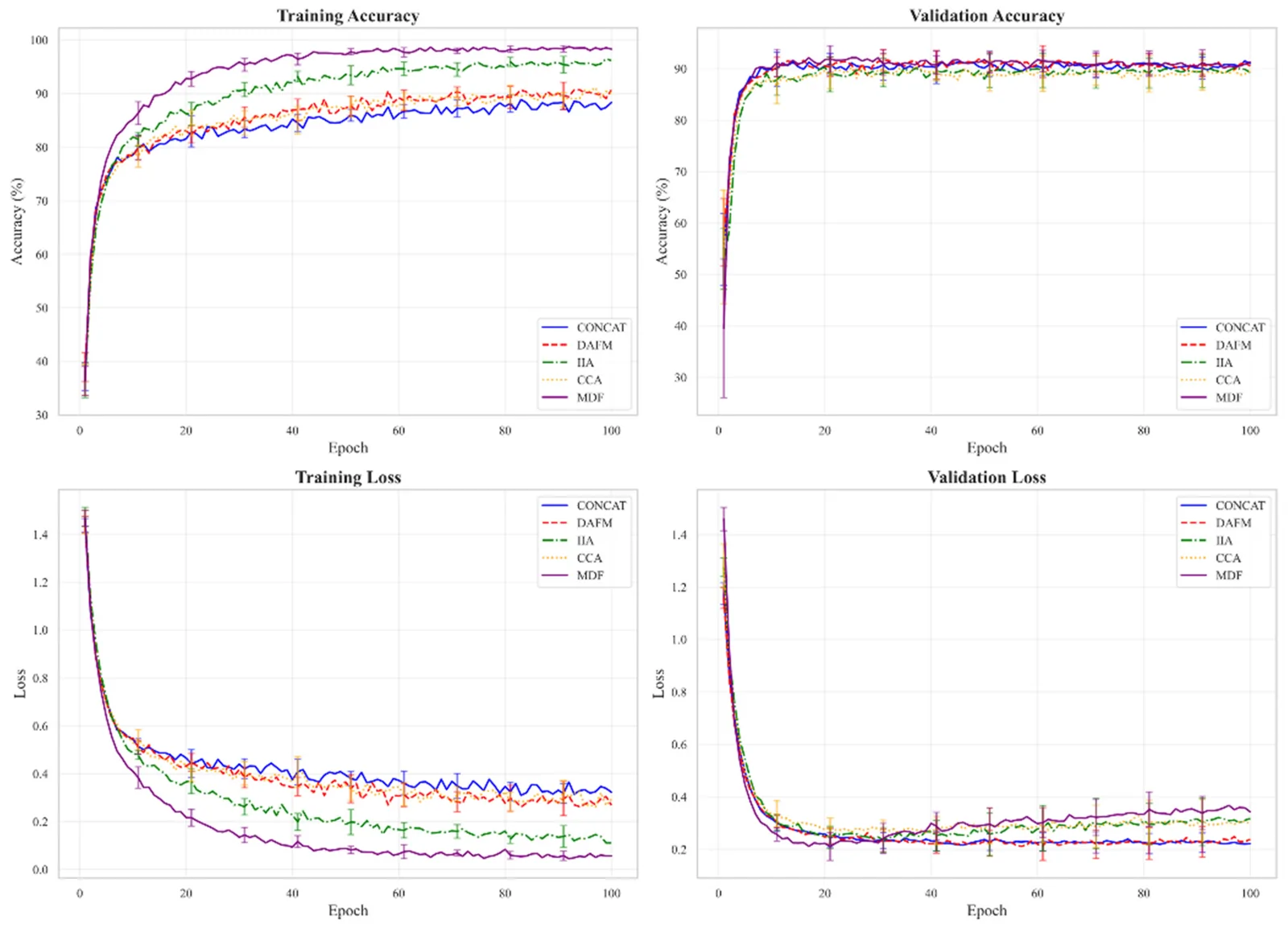
Training and validation behavior of different fusion mechanisms at 0 dB SNR.

**Figure 11:**
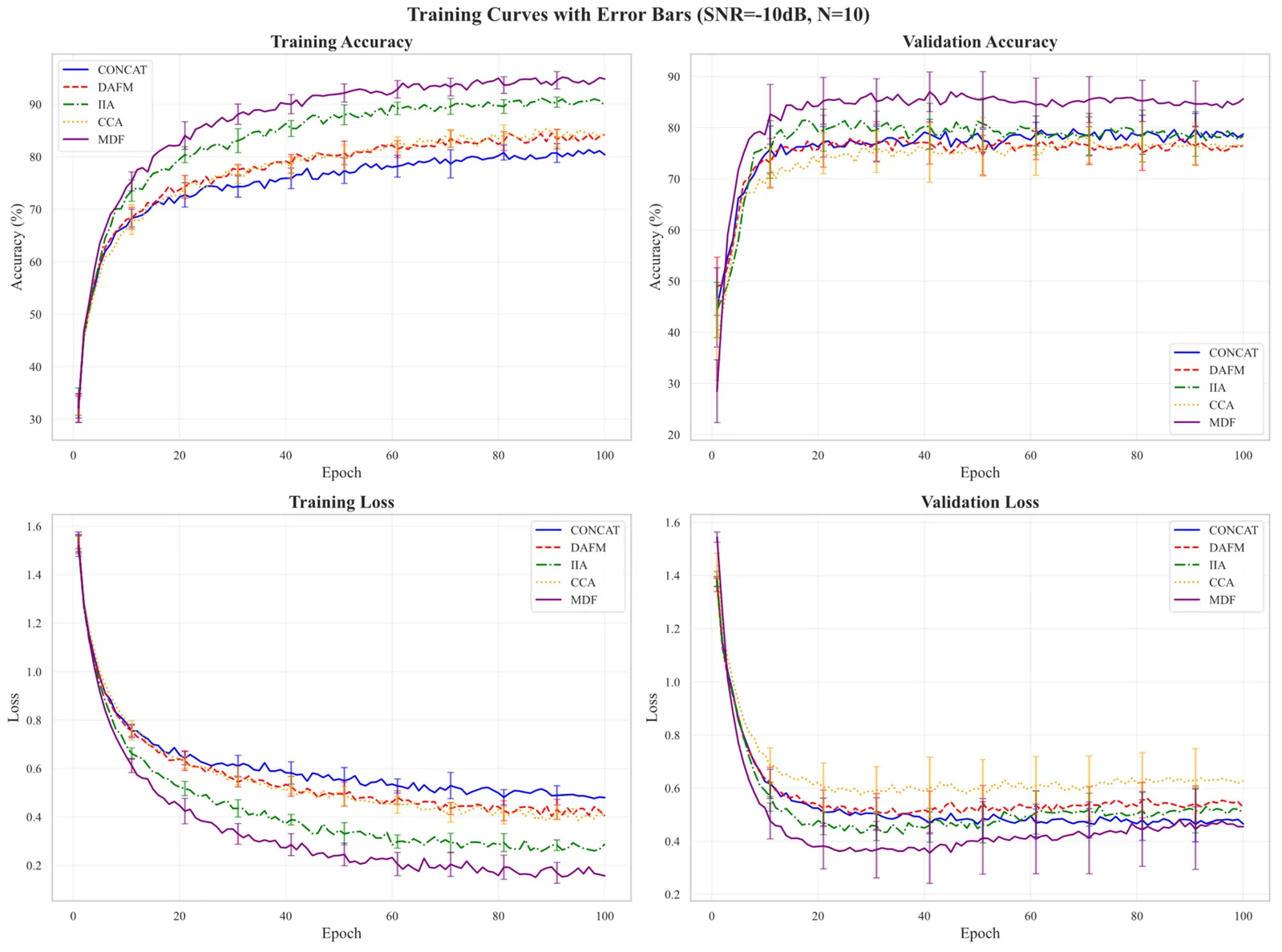
Training and validation behavior of different fusion mechanisms at −10 dB SNR.

To further investigate the influence of noise on the optimization process, Figure 10 and Figure 11 compare the training and validation behavior of the five fusion mechanisms at 0 dB and −10 dB SNR, respectively. At 0 dB SNR, all fusion mechanisms can generally achieve stable convergence, and the differences in final validation performance are relatively small. Under the more severe −10 dB condition, however, the weaker fusion strategies exhibit larger fluctuations, slower convergence, or lower validation accuracy. In comparison, MDF retains relatively stable optimization behavior and a higher validation level, indicating that its robustness advantage is not limited to the final test accuracy but is also reflected in the learning dynamics under strong noise interference.

**Figure 12:**
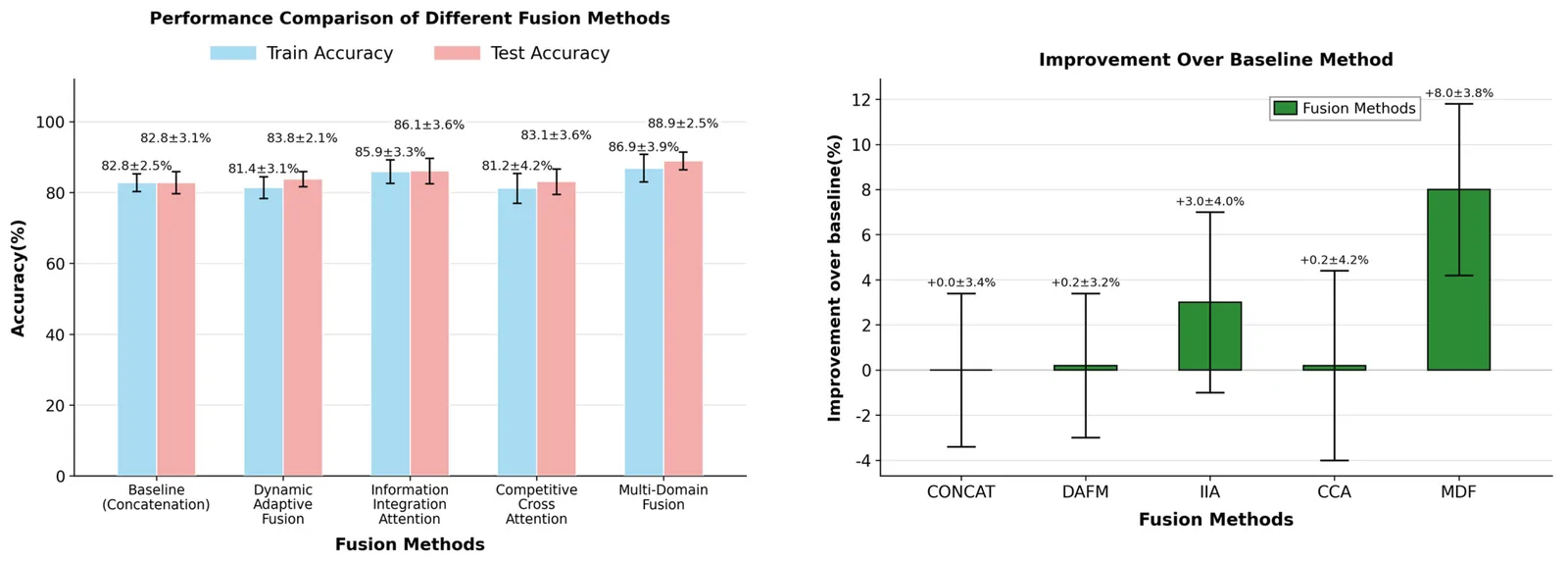
Performance comparison of five fusion methods at −10 dB SNR.

A more direct comparison under the harshest noise condition is provided in Figure 12. At −10 dB SNR, MDF achieves the highest test accuracy among the five fusion methods, while IIA provides the second-best performance. In contrast, CONCAT, DAFM, and CCA exhibit larger performance degradation. This result is consistent with the fitted degradation rates obtained from Figure 9 and Table 5, further confirming that the fusion strategy itself plays an important role in maintaining diagnostic reliability under noisy conditions.

**Figure 13:**
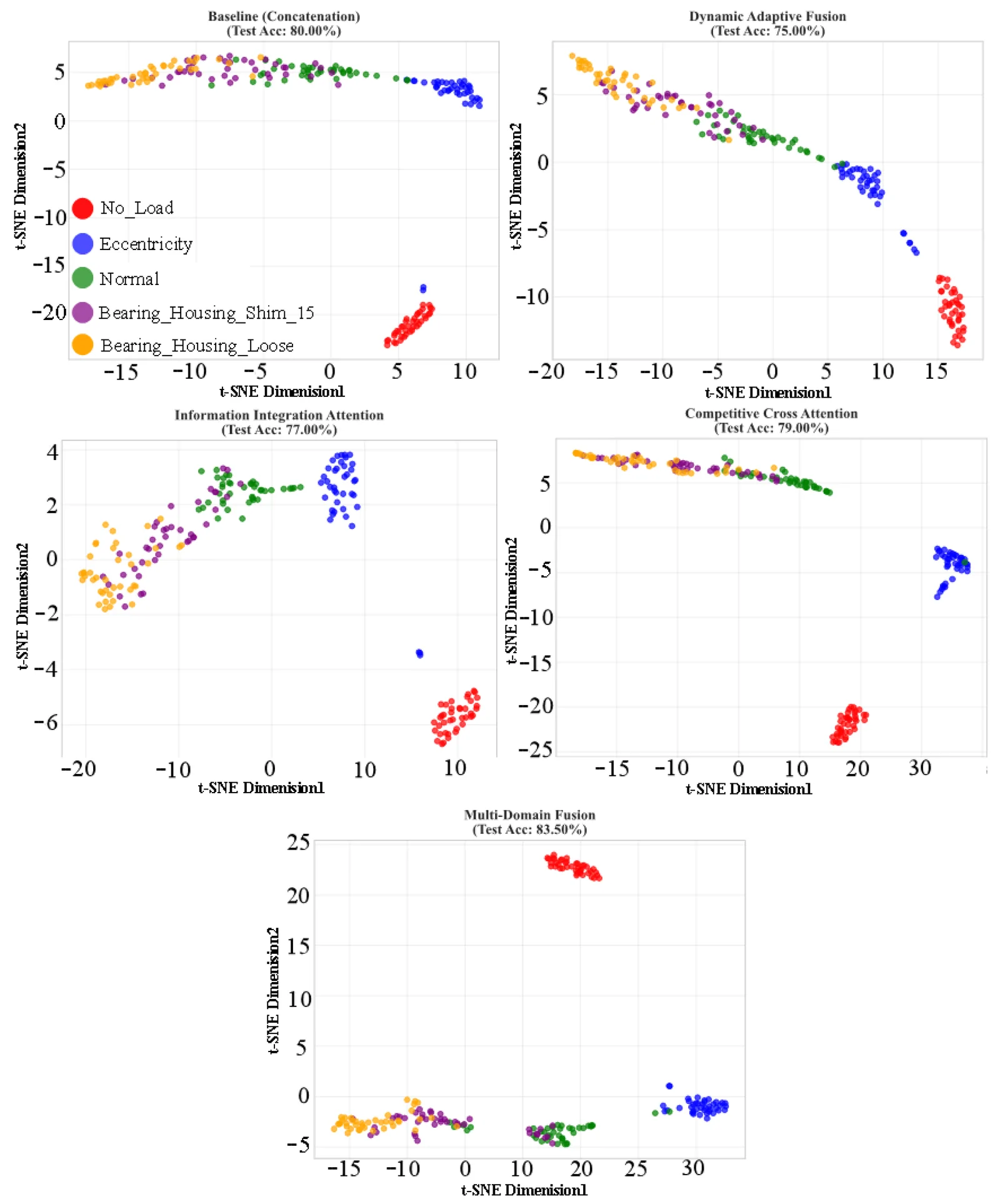
T-SNE visualization of the fused feature representations obtained by the five fusion mechanisms at −10 dB SNR.

The representational basis of the robustness advantage is further illustrated in Figure 13, which presents the T-SNE visualization of the fused feature spaces at −10 dB SNR. Under severe noise interference, the feature distributions produced by simpler fusion methods show greater overlap among several machine states. In contrast, the MDF representation maintains more compact intra-class clusters and clearer inter-class boundaries. This observation suggests that MDF is better able to preserve state-discriminative information and suppress noise-sensitive components, thereby producing a more stable latent representation for downstream classification.

Taken together, the evidence from Figure 9, Table 4 and Table 5, and Figure 10, Figure 11, Figure 12 and Figure 13 provides a consistent answer to RQ3. The robustness of the diagnosis framework depends strongly on the adopted fusion mechanism. Among the evaluated strategies, MDF exhibits the smallest fitted degradation rate, the smallest overall accuracy loss from 0 to −10 dB, the highest test accuracy under severe noise, and the clearest class separation in the fused feature space. Therefore, MDF provides the strongest overall noise robustness among the evaluated fusion mechanisms.

### 4.4. Discussion, Practical Implications, and Validation Scope

The results presented in Section 4.1, Section 4.2 and Section 4.3 provide complementary answers to the three research questions formulated in Section 1.3. For RQ1, multi-direction feature fusion substantially improves machine-state recognition compared with individual sensing directions. The best single-direction accuracy of 86.00% is increased to 96.00% after fusion, corresponding to a 10.00-percentage-point improvement and an approximately 71.4% relative reduction in classification error. For RQ2, although CNN achieves the highest test accuracy of 98.00%, the lightweight MLP reaches 96.00% with substantially lower model complexity, demonstrating a more favorable balance between diagnostic performance and computational efficiency. For RQ3, the robustness analysis shows that MDF exhibits the slowest degradation under increasing noise, with a fitted degradation rate of 0.636 percentage points/dB and an accuracy of 88.88 ± 2.53% at −10 dB SNR. Taken together, these results indicate that the effectiveness of the proposed framework arises from the coordinated design of feature representation, feature selection, fusion, and lightweight classification rather than from increasing network complexity alone.

From a methodological perspective, the results suggest that the quality of the input representation plays an important role in the present diagnosis task. Before classification, the three-direction vibration signals are transformed into complementary time-, frequency-, and wavelet-domain descriptors and subsequently refined through random-forest-based supervised feature selection. The original 231 handcrafted features are reduced to 60 selected features before fusion, thereby removing substantial redundancy while retaining diagnostically relevant information. As a result, the downstream classifier operates on a compact and highly informative representation rather than directly learning discriminative characteristics from raw vibration signals. This helps explain why a lightweight fully connected network remains competitive with structurally more complex CNN-, MLP-Mixer-, and Transformer-based classifiers in the current experimental setting.

The proposed framework also has several practical implications for deployment-oriented machine monitoring. First, the X-, Y-, and Z-direction vibration signals are acquired simultaneously using a single three-axis piezoelectric accelerometer, so the performance gain obtained from multi-direction fusion does not require three independently installed vibration sensors or additional sequential acquisition time. Second, random-forest-based feature selection reduces the initial feature dimension from 231 to 60 before fusion, limiting the computational burden introduced by multi-direction processing. Third, the use of a lightweight MLP further reduces downstream inference complexity. These characteristics make the framework potentially suitable for resource-constrained monitoring systems. Nevertheless, deployment suitability in this study is evaluated primarily from algorithmic complexity rather than from direct measurements of end-to-end latency, memory consumption, energy usage, or throughput on a specific industrial edge device. These hardware-level deployment metrics therefore remain to be experimentally validated.

The experimental scope should also be interpreted carefully. The five machine states considered in this study were evaluated on a controlled milling-machine platform under predefined operating conditions. This setting was intentionally adopted to isolate the effects of sensing direction, feature representation, classifier architecture, fusion strategy, and signal degradation, thereby enabling reproducible and internally consistent comparisons across the experiments. Controlled experimental validation is therefore an appropriate stage for establishing the methodological effectiveness of the proposed framework rather than, by itself, evidence of universal generalization to all industrial operating conditions. Similar controlled platforms and predefined operating scenarios have also been widely adopted in milling-machine and multi-sensor monitoring studies before broader industrial validation [3,8].

A similar interpretation applies to the noise-robustness experiments. Additive Gaussian white noise was introduced as a controlled perturbation because its intensity can be explicitly specified through the signal-to-noise ratio, allowing different fusion mechanisms to be compared under reproducible levels of signal degradation. Such SNR-based noise injection is widely used as a standardized stress-test protocol in machinery fault-diagnosis research. For example, Cao and Zhang evaluated a multi-domain denoising diagnosis framework using Gaussian white noise over SNR levels from 0 to −10 dB and reported accuracies of 81.25% and 76.36% at −10 dB on the CWRU and MFPT datasets, respectively [39]. Lin et al. similarly investigated a lightweight anti-noise diagnostic framework over the −10 to 0 dB range and reported an accuracy of 89.42% at −10 dB on the RABB2 dataset [40]. These numerical results are provided only as methodological context rather than as direct performance benchmarks, because the datasets, machine types, state definitions, sensing conditions, and model objectives differ substantially across studies.

Accordingly, the Gaussian-noise experiments in the present study should be regarded as a standardized evaluation of relative noise robustness rather than a complete reproduction of real industrial disturbance. Actual machine-tool environments may involve non-stationary cutting excitation, structural vibration transmitted from adjacent components or equipment, periodic mechanical interference, electromagnetic disturbance, sensor-mounting variation, changes in spindle speed and feed rate, and combinations of multiple noise sources. The complex and coupled nature of vibration and noise in machine tools has been extensively discussed in the literature [4]. Future robustness evaluation should therefore incorporate measured background noise from practical machining environments, non-Gaussian and mixed interference, and variable operating conditions to determine whether the relative advantages observed under controlled SNR degradation remain valid in realistic industrial scenarios.

Several additional aspects also define the present validation scope. The current framework uses a fixed sensing configuration and focuses primarily on offline machine-state recognition. Its generalization across different machines, machining parameters, sensor installations, and previously unseen operating conditions has not yet been systematically evaluated. Furthermore, practical long-term monitoring may involve sensor degradation, missing channels, distribution shifts, and previously unseen machine states. Future work will therefore investigate online adaptation, sensor-fault tolerance, cross-condition and cross-machine transfer learning, open-set recognition, and integration of additional sensing modalities [41]. The framework will also be implemented on practical edge-computing hardware to evaluate inference latency, memory footprint, power consumption, and long-term operational stability under real machining conditions.

### 4.5. Component-Level Ablation Study of MDF

Although Section 4.1, Section 4.2 and Section 4.3 evaluate multi-directional feature fusion, classifier architectures, and different fusion mechanisms, these comparisons do not independently isolate the contributions of the internal components of MDF. In addition, CONCAT, DAFM, IIA, and CCA primarily perform information integration along the sensing-direction axis, whereas MDF further reorganizes the selected features according to the time, frequency, and wavelet domains. Therefore, a dedicated component-level ablation study was conducted to (1) quantify the contributions of domain-specific encoding and adaptive domain attention and (2) provide a controlled same-axis baseline for MDF.

Three configurations were evaluated. The first configuration, Global Projection, removes both the domain-specific encoding structure and adaptive domain attention. After random-forest-based feature selection, the 60 selected features are directly combined and mapped into a common 32-dimensional latent representation using a single fully connected layer followed by ReLU:

(25) Fd=ReLU(WdFdin+bd),d∈time,freq,wavelet

where **F***_d_* ∈ ℝ^60^, **W***_d_* ∈ ℝ^32×60^, and b*_d_* ∈ ℝ^32^. This configuration provides a baseline for evaluating whether explicitly preserving the feature-domain structure is beneficial.

The second configuration, Domain Encoding + Uniform Fusion, retains exactly the same time-, frequency-, and wavelet-domain encoders defined for MDF in Equations (12)–(14). However, the adaptive attention mechanism in Equations (15) and (16) is removed. Instead, the three 32-dimensional domain representations are combined using fixed equal weights:

(26) $$F_{\mathrm{uniform}}=\frac{1}{3}(F_{\mathrm{time}}+F_{\mathrm{freq}}+F_{\mathrm{wavelet}})$$

This configuration serves as a controlled same-axis baseline for MDF. Both Domain Encoding + Uniform Fusion and Full MDF operate on the same time-, frequency-, and wavelet-domain representations, use the same domain-specific encoders, generate the same 32-dimensional fused representation, and employ the identical downstream MLP classifier. Their principal difference is therefore the fusion rule: the former uses fixed equal domain weights, whereas Full MDF uses the input-dependent adaptive domain-attention mechanism defined in Equations (15) and (16).

The third configuration is the Full MDF described in Section 2.4. No modification is made to its architecture or training procedure. To reduce the potential influence of model capacity, all three configurations produce a 32-dimensional output and use the same downstream classifier, dataset partition, and optimization protocol. Their fusion modules contain approximately 1.95 K, 2.02 K, and 2.12 K trainable parameters for Global Projection, Domain Encoding + Uniform Fusion, and Full MDF, respectively. Thus, the three configurations have highly comparable parameter scales.

Because MDF is primarily intended to improve information integration under degraded signal conditions, the component-level ablation was evaluated at both 0 dB and −10 dB SNR. The results are summarized in Table 6.

As shown in Table 6, at 0 dB SNR, Global Projection achieves an accuracy of 81.10 ± 1.32%. Introducing domain-specific encoding while retaining uniform fusion increases the accuracy to 86.70 ± 1.04%, corresponding to an improvement of 5.60 percentage points. This result indicates that explicitly preserving the structural differences among time-, frequency-, and wavelet-domain features produces a more discriminative representation than directly projecting all selected features into a single latent space.

When adaptive domain attention is further introduced, the accuracy increases from 86.70 ± 1.04% to 94.38 ± 1.96%, providing an additional improvement of 7.68 percentage points. Therefore, domain-specific encoding alone does not account for the full performance advantage of MDF; dynamically adjusting the relative contributions of the three feature domains provides substantial additional benefit.

The same pattern is observed under the more severe −10 dB condition. Global Projection achieves 76.30 ± 2.02%, while Domain Encoding + Uniform Fusion reaches 81.60 ± 1.61%, corresponding to an improvement of 5.30 percentage points. Full MDF further increases the accuracy to 88.88 ± 2.53%, yielding an additional improvement of 7.28 percentage points over uniform fusion. These results demonstrate that the contributions of both domain-specific encoding and adaptive domain attention remain evident under severe signal degradation.

More importantly, the comparison between Domain Encoding + Uniform Fusion and Full MDF provides a direct same-axis comparison. Both configurations operate on exactly the same time-, frequency-, and wavelet-domain representations and differ primarily in whether uniform or adaptive domain weighting is employed. MDF improves accuracy over this same-axis baseline by 7.68 percentage points at 0 dB and 7.28 percentage points at −10 dB. Therefore, the performance advantage of MDF cannot be attributed solely to changing the fusion axis from sensing directions to feature domains; the adaptive domain-weighting mechanism itself provides a clear additional contribution.

Overall, Full MDF outperforms Global Projection by 13.28 percentage points at 0 dB and 12.58 percentage points at −10 dB. Because the three configurations have similar parameter counts, identical output dimensionality, and the same downstream classifier and optimization protocol, the observed differences are unlikely to be explained simply by increased model capacity. Instead, the results support the complementary roles of domain-specific representation learning and adaptive domain weighting in the proposed MDF mechanism.

Taken together, the ablation study provides two complementary findings. First, separating heterogeneous features according to their physical domains improves the quality of the learned representation. Second, adaptive weighting further enhances the utilization of these domain-specific representations compared with fixed equal weighting. These findings complement the cross-method robustness comparison in Section 4.3 and provide direct component-level evidence for the effectiveness of MDF.

## 5. Conclusions

This paper presented a multi-sensor feature-level fusion framework for fault diagnosis in high-precision milling machines, with particular emphasis on diagnostic accuracy, computational efficiency, and deployment suitability in industrial environments. The proposed framework integrates multi-direction vibration acquisition, multi-domain feature construction, random-forest-based feature selection, feature-level fusion, and lightweight neural classification into a unified diagnostic pipeline.

The experimental results provide clear answers to the three research questions addressed in this study. First, RQ1 shows that multi-directional vibration feature fusion improves diagnostic accuracy from 74.00–86.00% for individual sensing directions to 96.00%, while the additional burden is mainly associated with increased feature-processing dimensionality rather than additional sensing time, owing to simultaneous three-axis acquisition. Second, RQ2 shows that the lightweight MLP provides the most favorable trade-off between diagnostic performance and computational efficiency among the evaluated classifier backbones, and is therefore selected as the default classifier in the proposed framework. Third, RQ3 demonstrates that robustness depends strongly on the fusion mechanism, and that MDF is the most reliable strategy for preserving diagnostic performance under severe noise interference.

Overall, the present study demonstrates that accurate, efficient, and noise-robust milling-machine fault diagnosis can be achieved through a coordinated combination of multi-sensor feature fusion, supervised feature selection, lightweight MLP classification, and MDF-based robust fusion. Future work will extend this framework to more diverse operating conditions, additional sensing modalities, online diagnosis, and cross-machine transferability, and will further validate its deployment capability on practical edge devices.

## Figures and Tables

**Table 1:** Dataset Composition and Record-Level Partition for the Five Machine States.

Label	Machine State	Description	Raw Records	Training Records	Validation Records	Test Records
C1	No_Load	Spindle running without machining load (non-fault operating state)	10	7	1	2
C2	Eccentricity	Eccentricity-related abnormal operating state	10	7	1	2
C3	Normal	Normal machining operating state	10	7	1	2
C4	Bearing_Housing_Shim_15	Abnormal state induced by inserting a 0.15 mm copper shim at the bearing housing	10	7	1	2
C5	Bearing_Housing_Loose	Abnormal state induced by loosening the bearing housing	10	7	1	2
Total	—	—	50	35	5	10

**Table 2:** Training Hyperparameters and Lightweight MLP Configuration.

Parameter	Value	Purpose/Rationale
Optimizer	AdamW	Stable optimization with decoupled weight decay
Initial learning rate	0.001	Moderate update magnitude for stable convergence
Weight decay	0.01	Regularization to reduce overfitting
Batch size	32	Balance between gradient stability and computational efficiency
Maximum epochs	100	Common training horizon for comparing optimization and generalization behavior
Record-level train/validation/test split	70%/10%/20%	Consistent and leakage-free dataset partition
Dropout rate	0.3	Reduces co-adaptation and overfitting
Label smoothing	0.05	Reduces overconfident predictions
Gradient clipping	Max norm = 1.0	Suppresses excessively large gradient updates
LR scheduler	ReduceLROnPlateau	Adaptive learning-rate adjustment
LR reduction factor	0.5	Halves learning rate after stagnation
Scheduler patience	8 epochs	Avoids premature LR reduction
Minimum learning rate	1 × 10^5^	Prevents excessively small updates

**Table 3:** Quantitative training and convergence characteristics of the single-direction and fused-feature models.

Model	Final Training Loss	Final Validation Loss	Absolute Loss Gap	Last-10-Epoch Training-Loss Change (%)
X-direction	0.52	0.69	0.17	7.8
Y-direction	0.57	0.68	0.11	4.3
Z-direction	0.54	0.77	0.23	5.3
Multi-direction Fusion	0.49	0.46	0.03	2.5

**Table 4:** Test Accuracy (%) of Different Fusion Mechanisms under Different SNR Levels over 10 Independent Runs.

SNR	CONCAT	DAFM	IIA	CCA	MDF
0	94.12 ± 1.86%	94.50 ± 1.39%	93.12 ± 1.61%	92.75 ± 1.66%	94.38 ± 1.96%
−2	91.62 ± 2.68%	92.62 ± 2.05%	92.25 ± 3.20%	90.25 ± 4.21%	92.88 ± 2.68%
−4	88.38 ± 3.06%	88.00 ± 2.86%	89.75 ± 1.56%	87.12 ± 3.26%	90.62 ± 2.86%
−6	86.00 ± 4.47%	86.25 ± 4.26%	89.50 ± 3.54%	87.25 ± 4.02%	89.38 ± 3.07%
−8	82.50 ± 2.44%	82.12 ± 2.10%	86.75 ± 2.11%	84.00 ± 3.70%	87.62 ± 4.31%
−10	82.75 ± 3.10%	83.75 ± 2.09%	86.12 ± 3.64%	83.12 ± 3.59%	88.88 ± 2.53%

**Table 5:** Linear-Fitting Results for Accuracy Degradation Under Increasing Noise.

Fusion Method	Fitted Relationship A(D)=b−kD	Degradation Rate k (pp/dB)	$R^{2}$	Accuracy Drop from 0 to −10 dB (pp)
CONCAT	*A* = 93.747 − 1.237*D*	1.237	0.962	11.37
DAFM	*A* = 94.088 − 1.243*D*	1.243	0.907	10.75
IIA	*A* = 93.278 − 0.739*D*	0.739	0.964	7.00
CCA	*A* = 92.184 − 0.954*D*	0.954	0.955	9.63
MDF	*A* = 93.807 − 0.636*D*	0.636	0.863	5.50

**Table 6:** Component-Level Ablation Results of MDF under Different Noise Conditions.

Configuration	Domain-Specific Encoding	Adaptive Domain Attention	Parameters	0 dB Accuracy (%)	−10 dB Accuracy (%)
Global Projection	No	No	1.95 K	81.10 ± 1.32	76.30 ± 2.02
Domain Encoding + Uniform Fusion	Yes	No	2.02 K	86.70 ± 1.04	81.60 ± 1.61
Full MDF	Yes	Yes	2.12 K	94.38 ± 1.96	88.88 ± 2.53

## Data Availability

The data presented in this study are available from the corresponding author upon reasonable request.
